# On the correlation between hydrogen bonding and melting points in the inositols

**DOI:** 10.1107/S2052252513026511

**Published:** 2013-10-18

**Authors:** Sándor L. Bekö, Edith Alig, Martin U. Schmidt, Jacco van de Streek

**Affiliations:** a Institute for Inorganic and Analytical Chemistry, Goethe-University, Max-von-Laue-Str. 7, 60438 Frankfurt am Main, Germany; b Department of Pharmacy, University of Copenhagen, Universitetsparken 2, 2100 Copenhagen, Denmark

**Keywords:** inositol, X-ray powder diffraction, melting point, rotator phase, polymorphism

## Abstract

Inositol, 1,2,3,4,5,6-hexahydroxycyclohexane, exists in nine stereoisomers with different crystal structures and melting points. In a previous paper on the relationship between the melting points of the inositols and the hydrogen-bonding patterns in their crystal structures [Simperler *et al.* (2006 ▶). *CrystEngComm* **8**, 589], it was noted that although all inositol crystal structures known at that time contained 12 hydrogen bonds per molecule, their melting points span a large range of about 170 °C. Our preliminary investigations suggested that the highest melting point must be corrected for the effect of molecular symmetry, and that the three lowest melting points may need to be revised. This prompted a full investigation, with additional experiments on six of the nine inositols. Thirteen new phases were discovered; for all of these their crystal structures were examined. The crystal structures of eight ordered phases could be determined, of which seven were obtained from laboratory X-ray powder diffraction data. Five additional phases turned out to be rotator phases and only their unit cells could be determined. Two previously unknown melting points were measured, as well as most enthalpies of melting. Several previously reported melting points were shown to be solid-to-solid phase transitions or decomposition points. Our experiments have revealed a complex picture of phases, rotator phases and phase transitions, in which a simple correlation between melting points and hydrogen-bonding patterns is not feasible.

## Abstract 2

13 new phases of the inositols, 1,2,3,4,5,6-hexahydroxycyclohexane, were found. Crystal structure determinations and thermal analyses reveal a very complex picture of phases, rotator phases and phase transitions.

## 1. Introduction   

**Figure 1:**
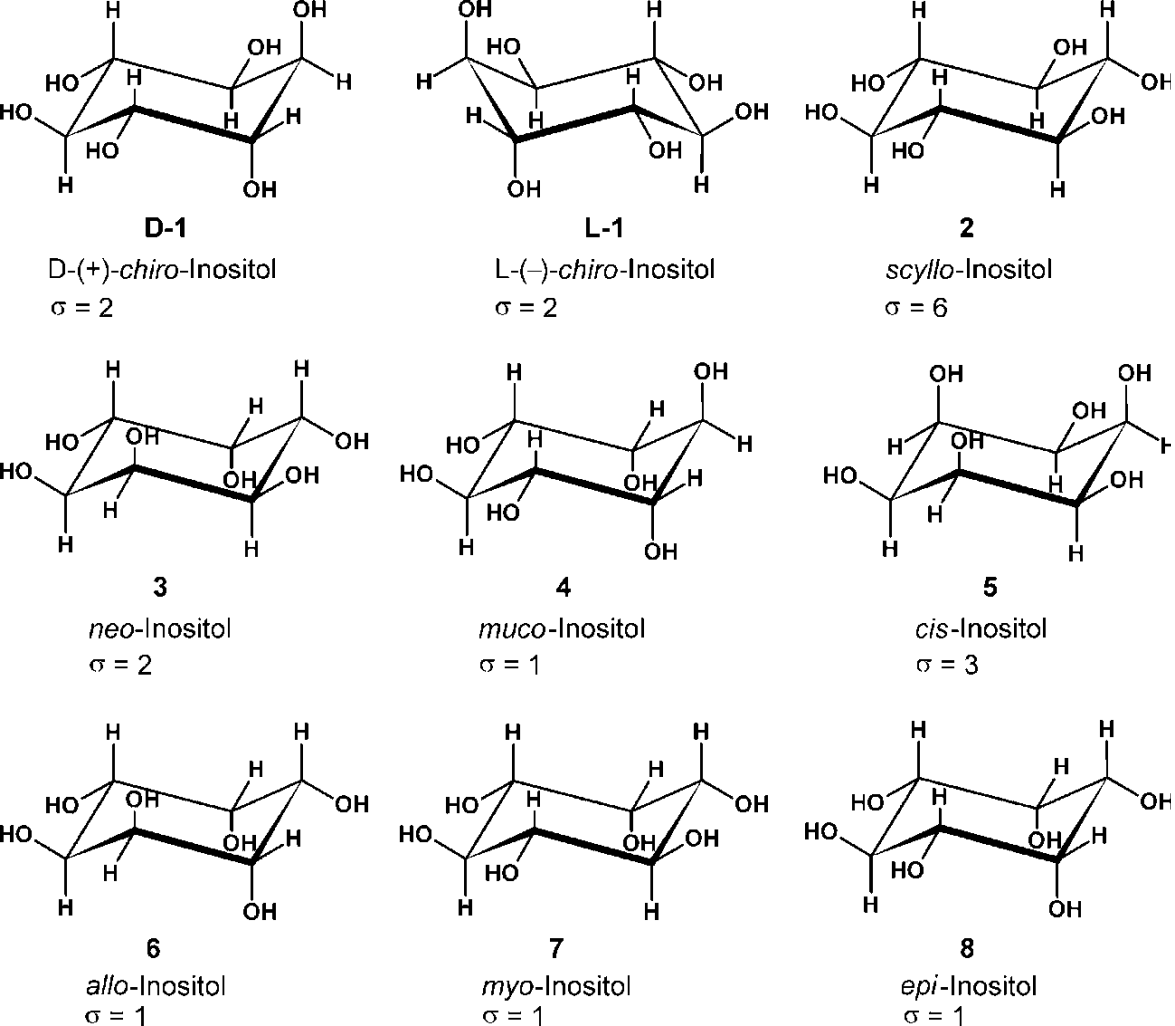
Inositol stereoisomers and molecular symmetry numbers σ.

The term *inositol*, 1,2,3,4,5,6-hexahydroxycyclohexane, denotes a class of compounds whose basis is provided by the nine stereoisomers in Fig. 1 ▶ (for the nomenclature and numbering of cyclitoles, refer to Dawson *et al.*, 1973 ▶; Parthasarathy & Eisenberg, 1991 ▶). All inositol isomers exhibit the same chemical composition, C_6_H_12_O_6_, but each of them with its own configuration. Four of them [*myo*-, *scyllo*-, d-(+)-*chiro* and l-(−)-*chiro*-inositol] occur in nature, the remaining five (*cis*-, *epi*-, *allo*-, *neo*- and *muco*-inositol) have synthetic origins. All of them could be synthesized and described in the past and their syntheses optimized in recent years (Posternak, 1951 ▶; Angyal, 1957 ▶; Angyal & McHugh, 1957 ▶; Angyal & Hickman, 1971 ▶; Angyal *et al.*, 1995 ▶; Chung & Kwon, 1999 ▶). d-(+)-*chiro*- and l-(−)-*chiro*-inositol are enantiomers, and their crystal structures can be expected to be mirror images, with identical thermodynamic properties such as melting points.

Our interest in the inositols was sparked by a paper by Simperler *et al.* (2006 ▶) concerning the correlation of the melting points of the inositols with the hydrogen-bonding patterns in their crystal structures. In all the inositol crystal structures known at that time, each inositol molecule was connected to its neighbours by 12 hydrogen bonds. Based on the simple criterion of counting hydrogen bonds, the melting points would therefore be expected to be fairly similar. Surprisingly, the melting points reported in the paper by Simperler *et al.* span a large range from 180 to 350 °C. In particular, *scyllo*-inositol had a significantly higher melting point than the remaining inositols, whereas the melting point of *allo*-inositol was significantly lower. We noticed that the explanations for each of these two anomalous melting points could be found in the literature.

The excellent paper by Wei (1999 ▶) describes how high molecular symmetry gives rise to elevated melting points in homologous series of compounds. In brief, molecules of high point-group symmetry – high symmetry number, σ, to be precise – benefit less from the rotational degrees of freedom that become available upon melting, and as such resist melting and have higher melting points; it is an entropy effect that follows from statistical thermodynamics. The symmetry number σ corresponds to the order of the point group if only proper rotations and the identity are counted. Wei’s paper offers an explanation and quantification of Carnelley’s rule, published in 1882 (Carnelley, 1882*a* ▶,*b* ▶). The remarkable melting point behaviour observed in other series of isomeric or homologous compounds (Joseph *et al.*, 2011 ▶; Podsiadło *et al.*, 2012 ▶) may also be explained by this effect.

All inositols in the Simperler paper have σ = 1 or σ = 2, with the exception of *scyllo*-inositol, which has σ = 6. The connection between the high melting point of *scyllo*-inositol and its high molecular symmetry was mentioned earlier by Orloff (1954 ▶). The higher melting point of *scyllo*-inositol is therefore as expected based on its higher molecular symmetry. Calculation of the corrected melting point – the melting point *scyllo*-inositol would have in the absence of molecular symmetry – requires the value of the enthalpy of melting, *H*_m_.

The significantly lower melting point of *allo*-inositol can also be explained: in the paper that reports the crystal structure and its melting point of 180 °C (Bonnet *et al.*, 2006*a* ▶), another paper is cited that reports a melting point of 310 °C for *allo*-inositol (Tschamber *et al.*, 1992 ▶), essentially the same as *neo*- (315 °C) and *epi*-inositol (304 °C). It appears that *allo*-inositol exhibits polymorphism, and the change at 180 °C may well refer to a phase transition to another polymorph rather than to a melting point.

After *allo*-inositol, the second lowest melting point in the Simperler paper was reported for *myo*-inositol, at 225 °C. Interestingly, 1 year after the Simperler paper, Khan *et al.* (2007 ▶) reported a new polymorph for *myo*-inositol, with unknown melting point. This leaves room for speculation that perhaps the new polymorph has a higher melting point.

That would leave l-(−)-*chiro*-inositol and its enantiomer d-(+)-*chiro*-inositol as the only remaining inositols with a slightly lower melting point than the other inositols. Because d-(+)- and l-(−)-*chiro*-inositol are the only inositols that are chiral, they are the only inositols that cannot pack in a space group with an inversion centre or a glide plane – two symmetry elements that are known to lead to efficient packing (Kitaigorodskii, 1961 ▶). It is therefore to be expected that a racemic mixture of l-(−)-*chiro*-inositol and d-(+)-*chiro*-inositol is able to crystallize in a structure with a more stable packing and it may therefore have a melting point that is more in line with the other inositols.

For *cis*-inositol, only the crystal structure of a monohydrate has been published (Freeman *et al.*, 1996 ▶); we are not aware of a published melting point for *cis*-inositol.

We therefore set out to fill these gaps. Specifically, we wanted to find the high-melting polymorph of *allo*-inositol, to determine *H*_m_ for *scyllo*-inositol (to calculate its corrected melting point), to determine the melting point of the second polymorph of *myo*-inositol and to determine the crystal structures and melting points of *rac*-*chiro*-inositol and *cis*-inositol.

## 2. Experimental   

### 2.1. Materials and crystallization   

We denote the compounds by numbers (see Fig. 1 ▶) and the crystal phases by capital letters, *e.g.* **7**-A, **7**-B and **7**-C for the three polymorphs of *myo*-inositol.

d-(+)-*chiro*-Inositol (d-**1**·1/3H_2_O), l-(−)-*chiro*-inositol (l-**1**·1/3H_2_O), *cis*-inositol (**5**), *allo*-inositol (**6**) and *myo*-inositol (**7**) were purchased from Sigma Aldrich (≥ 98.0%), whereas *scyllo*-inositol (**2**) was purchased from TCI Europe (≥ 98.0%). All materials were used as received without further purification. The prices of the compounds allowed only small quantities to be purchased, which in turn hampered the growing of sizeable single crystals. The crystal structure determinations in this paper were therefore achieved using X-ray powder diffraction data, but the compounds are readily crystallized and for those phases stable at room temperature, single crystals can almost certainly be grown given sufficient starting material.

*rac*-*chiro*-Inositol (*rac*-**1**) was prepared by dissolving 30 mg of each enantiomer in 3 ml water. The solution was left to evaporate at room temperature and a white powder precipitated after *ca* 5 d.

### 2.2. X-ray powder diffraction (XRPD) and temperature-dependent X-ray powder diffraction (T-XRPD)   

Temperature-dependent X-ray powder diffraction data were recorded on a Stoe Stadi-P diffractometer with a Ge(111) monochromator (Cu *K*α_1_ radiation, λ = 1.5406 Å). For temperature regulation and detection, two different systems were used, depending on their application. For phase identification at temperatures up to 500 °C, a HUBER heater device 670.3 equipped with a high-temperature controller HTC 9634 and an imaging-plate position-sensitive detector (IP-PSD) were used. The heating rate was 5 °C min^−1^ for the mixture **5**-B + **5**-C, 3 °C min^−1^ for all other phases. Due to the limited 2θ range of 2–40° that is possible for this system, these measurements were not suitable for Pawley refinement or Rietveld refinement. Powder diffraction patterns for Pawley refinement (phases d-**1**-B, l-**1**-B, **5**-B and **6**-B) or Rietveld refinement (phases *rac*-**1**, d-**1**-A, **5**-A, **5**-D, **5**-E and **7**-C) were measured in transmission mode in a 0.7 mm diameter glass capillary from 2.0 to 80.0° in 2θ with 0.01° steps, using a linear position-sensitive detector and an Oxford Cryosystems 700 Series Cryostream, equipped with a Cryostream *Plus* controller. Each measurement lasted approximately 15 h. Compound **7**-C crystallizes in plates and was therefore additionally measured with amorphous SiO_2_ in a 2:1 ratio to minimize preferred orientation. The patterns were recorded at 25 (2) °C for *rac*-**1**, d-**1**-A, **5**-A, **5**-E and **7**-C, at 135 (2) °C for **5**-D, at 200 (2) °C for **5**-B and **6**-B, and at 227 (2) °C for d-**1**-B, l-**1**-B and the mixture of **5**-B + **5**-C. The software package *WinX^POW^* (Stoe & Cie, 2005 ▶) was used for data acquisition.

### 2.3. Structure determination from X-ray powder diffraction data   

The structure of d-**1**-A was derived from the known crystal structure of its enantiomer, l-**1**-A [Cambridge Structural Database (CSD; Allen, 2002 ▶) reference code FOPKOK, Jeffrey & Yeon, 1987 ▶]. The crystal structures of *rac*-**1**, **5**-A, **5**-D, **5**-E and **7**-C were solved from laboratory X-ray powder diffraction data using real-space methods within the program *DASH*3.1 (David *et al.*, 2006 ▶). The structures were subsequently refined by the Rietveld method using the program *TOPAS-Academic*4.1 (Coelho, 2007 ▶).

To aid the indexing process and the determination of the space group, the expected volume of an inositol molecule in the solid state was calculated by averaging the molecular volumes of all known inositol crystal structures that had been determined from single-crystal data, yielding 184 ± 5 Å^3^ at room temperature.

For indexing and structure solution, the powder patterns were truncated to a real-space resolution of about 2.5 Å. The backgrounds were subtracted with a Bayesian high-pass filter (David & Sivia, 2001 ▶). Peak positions for indexing were obtained by fitting approximately 20 manually selected peaks with an asymmetry-corrected full-Voigt function (Thompson *et al.*, 1987 ▶; Finger *et al.*, 1994 ▶). The powder patterns could be indexed with monoclinic lattices for *rac*-**1** and **5**-A and orthorhombic lattices for **5**-D, **5**-E and **7**-C without ambiguity using the program *DICVOL*91 (Boultif & Louër, 1991 ▶) with the corresponding figures of merit (de Wolff, 1968 ▶; Smith & Snyder, 1979 ▶) *M*(20) = 25.1 and *F*(20) = 57.0 for *rac*-**1**, *M*(20) = 45.8 and *F*(20) = 88.8 for **5**-A, *M*(17) = 39.1 and *F*(17) = 61.9 for **5**-D, *M*(20) = 25.5 and *F*(20) = 42.1 for **5**-E and *M*(20) = 35.8 and *F*(20) = 66.1 for **7**-C, and unit-cell volumes of 713.03 Å^3^ for *rac*-**1**, 743.02 Å^3^ for **5**-A, 1466.88 Å^3^ for **5**-D, 1442.97 Å^3^ for **5**-E and 721.24 Å^3^ for **7**-C after Pawley fit (Pawley, 1981 ▶). With an expected molecular volume of 184 Å^3^, these volumes correspond to 4, 4, 8, 8 and 4 molecules in the unit cell for *rac*-**1**, **5**-A, **5**-D, **5**-E and **7**-C, respectively. The close agreement of the indexed unit-cell volumes with the expected unit-cell volumes is another indication that the lattices did not contain further water or other solvent molecules. Using Bayesian statistical analysis (Markvardsen *et al.*, 2001 ▶), the space groups were determined to be *P*2_1_/*c* for *rac*-**1**, *P*2_1_/*n* for **5**-A, *Pbca* for **5**-D, *P*2_1_2_1_2_1_ for **5**-E and *Pca*2_1_ for **7**-C. Pawley refinements were then applied to extract integrated intensities and their correlations.

For structure solution, the starting molecular geometry for *rac*-**1** was taken from the single-crystal structure of the known polymorph of l-*chiro*-inositol (l-**1**-A) (CSD reference code FOPKOK; Jeffrey & Yeon, 1987 ▶), for **5**-A, **5**-D and **5**-E from *cis*-inositol monohydrate (**5**·H_2_O) (CSD reference code TAZMOW; Freeman *et al.*, 1996 ▶) and for **7**-C from polymorph B **7**-B (CSD reference code MYINOL01; Khan *et al.*, 2007 ▶). The crystal structures were solved without any problems.

After structure solution, Rietveld refinements were performed. All C atoms in each compound were assigned one global isotropic displacement parameter, as were all O atoms. The isotropic displacement parameter of the H atoms was constrained to be 1.2 times the global isotropic parameter of the parent atom. The preferred orientation in **7**-C could not be eliminated and a March–Dollase (Dollase, 1986 ▶) preferred orientation correction was therefore applied. A *Mogul* (Bruno *et al.*, 2004 ▶) geometry check of the refined crystal structures shows that all *z*-scores for all bond lengths and all angles are lower than 2.0.

The positions of the H atoms were determined by running short molecular dynamics simulations with the *COMPASS* force field (Sun, 1998 ▶) in *Materials Studio* (Accelrys, 2011 ▶) and quenching at regular intervals. The hydrogen-bonding pattern with the lowest energy was transferred to the experimental crystal structure and subsequently energy-minimized using dispersion-corrected density functional theory (Perdew *et al.*, 1996 ▶; Grimme, 2006 ▶), with the positions of the non-H atoms and the unit cell fixed. In *cis*-inositol monohydrate, single-crystal analysis showed the H atoms involved in intramolecular hydrogen bonds to be disordered (Freeman *et al.*, 1996 ▶). Our short molecular dynamics simulations show that it is highly likely that the H atoms involved in intramolecular hydrogen bonds in the high-temperature phase **5**-D, and probably also in **5**-E, are also disordered.

### 2.4. Thermal analysis (DSC and TGA)   

Differential scanning calorimetry (DSC) measurements were performed on a SETARAM (DSC 131) device. For each measurement, about 10 to 15 mg of the sample was filled into an Al crucible and measured at a rate of 1 °C min^−1^ under an N_2_ atmosphere. The given values for the temperatures are onset and offset values for the corresponding heating and cooling processes. Thermogravimetric analyses (TGA) were performed on a SETARAM (TGA 92) device. For each measurement, about 15 to 20 mg of the samples was filled into an Al_2_O_3_ (corundum) crucible and measured at a rate of 1 °C min^−1^ under an N_2_ atmosphere.

### 2.5. Elemental analysis (EA)   

Elemental analyses (CH) were carried out on an Elementar (vario MICRO cube) elemental analyzer. For each measurement, about 1 to 4 mg of the sample were placed into a Sn vessel and measured at 1150 °C under a He atmosphere with the addition of O_2_ during the measurement. The results are included in the supporting information.

## 3. Results and discussion   

### 3.1. Overview   

Thirteen new phases were found. The crystal structures of all eight ordered phases could be determined, of which seven were determined from laboratory X-ray powder diffraction data. The remaining five phases turned out to be rotator phases and only their unit cells could be determined. Melting points and phase-transition temperatures were recorded for investigated phases. An overview of the results is given in Tables 1 ▶ and 2 ▶.

### 3.2. *chiro*-Inositols (**1**)   

*chiro*-Inositol (**1**) exists in two enantiomers, d-(+)- and l-(−)-*chiro*-inositol. Both pure enantiomers and the racemate, *rac*-**1**, were investigated.

#### 3.2.1. d-(+)- and l-(−)-*chiro*-inositols   

**Figure 2:**
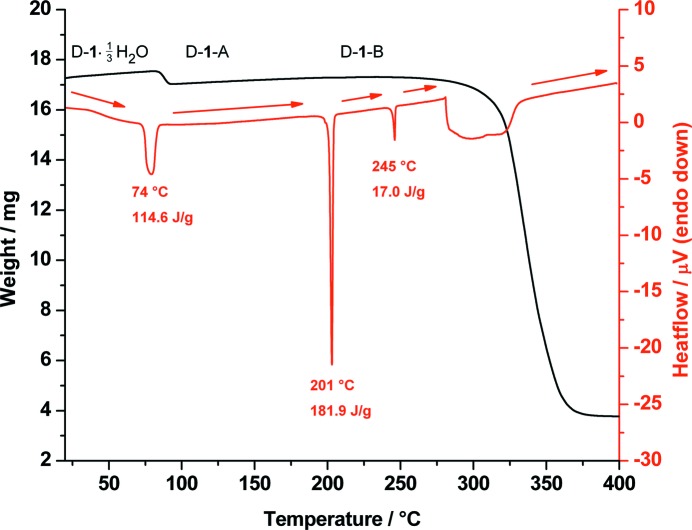
Combined DSC (red) and TGA (black) traces of the 1/3 hydrate of d-(+)-*chiro*-inositol (d-**1**·1/3H_2_O) measured from 20 to 400 °C.

The crystals initially obtained for d-(+)-*chiro*-inositol turned out to be a 1/3 hydrate, d-**1**·1/3H_2_O, as determined by single-crystal analysis. Hydrates are also known for *cis*-inositol (Freeman *et al.*, 1996 ▶) and for *myo*-inositol (Bonnet *et al.*, 2006*b* ▶; CSD reference code MYTOLD01). DSC analysis of d-**1**·1/3H_2_O shows a broad­ened endothermic signal with an onset at about 74 °C resulting from the loss of water and conversion of the 1/3 hydrate to the known anhydrate (d-**1**-A) (Jeffrey & Yeon, 1987 ▶). The TGA curve shows a mass loss of about 2.98% between 83 and 93 °C corresponding to a loss of approximately 0.3 water molecules per d-(+)-*chiro*-inositol molecule (Fig. 2 ▶).

In the DSC, three further endothermic signals could be observed; the first sharp peak at 201 °C resulting from a phase transition to the high-temperature polymorph, (d-**1**-B), the second sharp peak at 245 °C from melting and a third broad signal between 281 and 337 °C resulting from decomposition. The enthalpy of the phase transition at 201 °C is remarkably large, whereas the melting enthalpy at 245 °C is remarkably small. This is because the high-temperature phase (d-**1**-B) is a rotator phase (see §3.9) and the major part of the melting process takes place at 201 °C, with only the translational order of the centres of mass of the molecules remaining. This translational order is then lost when the final melting takes place at 245 °C.

**Figure 3:**
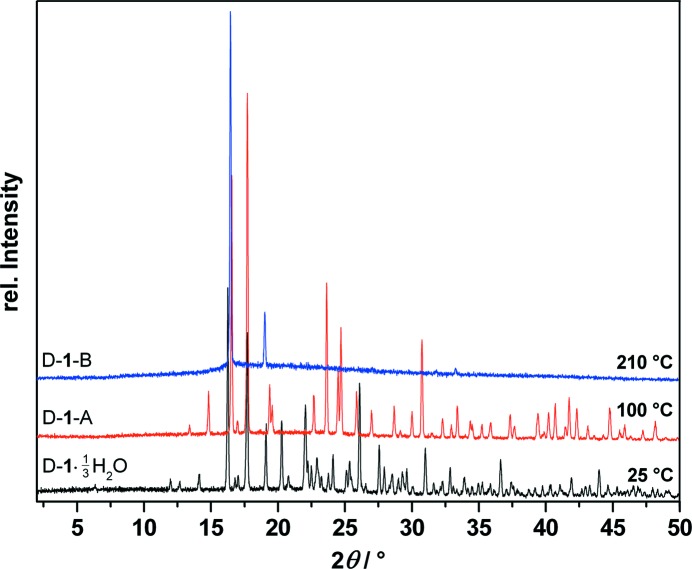
Temperature-dependent X-ray powder diffraction traces of d-(+)-*chiro*-inositol (d-**1**) at 25, 100 and 210 °C showing the phase transitions from the 1/3 hydrate (black) (d-**1**·1/3H_2_O) to the anhydrate (red) (d-**1**-A) and to the high-temperature polymorph (blue) (d-**1**-B).

The phases were identified by measuring T-XRPD patterns before and after the phase transitions (see Fig. 3 ▶).

The DSC and TGA curves and the XRPD patterns of l-**1** are the same as for its enantiomer d-**1**.

The crystal structures of the two 1/3 hydrates, l-**1**·1/3H_2_O and d-**1**·1/3H_2_O, will not be discussed in this paper, and this paper therefore only reports and discusses 11 of the 13 new phases.

The crystal structure of the room-temperature phase l-**1**-A was determined by Jeffrey & Yeon (1987 ▶). The enantiomeric crystal structure of d-**1**-A was established by Rietveld refinement (see the supporting information for full details). The molecules are connected to their neighbours by 12 hydrogen bonds (as determined with *Mercury*; Macrae *et al.*, 2008 ▶). Each —OH group acts as a donor and as an acceptor for one intermolecular hydrogen bond each, resulting in a three-dimensional network.

d-**1**-A does not rehydrate upon cooling to room temperature. The reversibility of the melting process and of the transition from **1**-A to **1**-B was not investigated. For structural investigations of the high-temperature rotator phases l-**1**-B and d-**1**-B, see §3.9.

#### 3.2.2. Racemic *chiro*-inositol   

**Figure 4:**
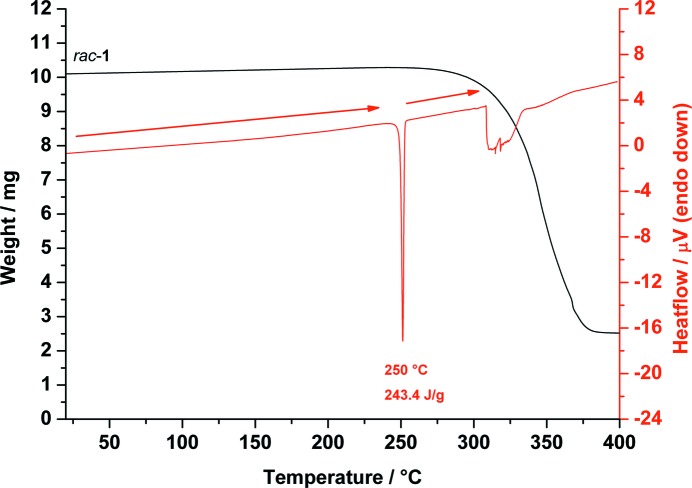
Combined DSC (red) and TGA (black) traces of *rac*-*chiro*-inositol (*rac*-**1**) measured from 20 to 400 °C showing the melting of *rac*-**1** at 250 °C.

The DSC analysis of *rac*-*chiro*-inositol, *rac*-**1**, shows only one endothermic signal at 250 °C from melting, which is 4–5 °C higher than for the pure enantiomers. Decomposition occurs as a broad signal between 308 and 344 °C. The TGA curve shows no mass loss before melting (see Fig. 4 ▶).

**Figure 5:**
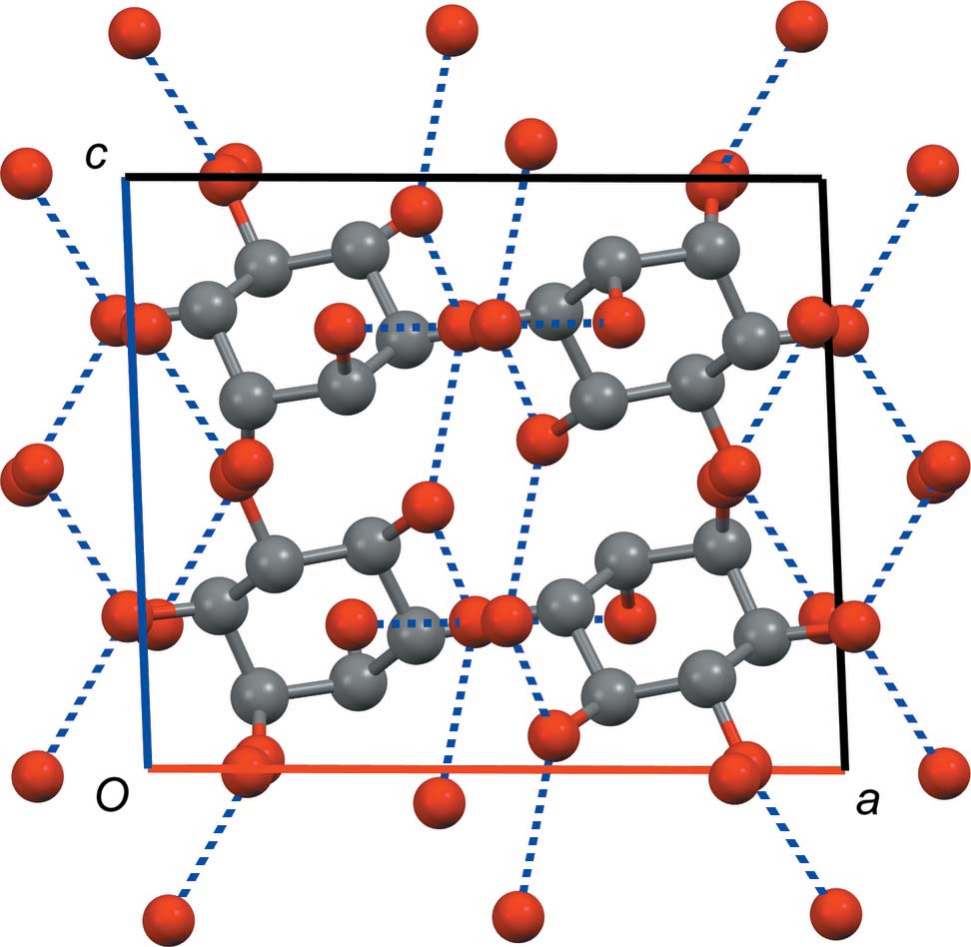
Crystal structure of racemic *chiro*-inositol (*rac*-**1**). Space group *P*2_1_/*c*, view along the *b* axis (*a* axis shown in red, *c* axis shown in blue). Hydrogen bonds are indicated as dashed blue lines, H atoms have been omitted for clarity.

The crystal structure of *rac*-**1** (see Fig. 5 ▶) was determined from powder diffraction data (the Rietveld refinement plot is shown in the supporting information). The compound crystallizes in the space group *P*2_1_/*c* with one molecule in the asymmetric unit. Each molecule is connected to the other molecules through 12 hydrogen bonds. In contrast to d-**1**-A and l-**1**-A, one O atom (O3) accepts two hydrogen bonds, while another (O2) accepts none.

### 3.3. *scyllo*-Inositol (**2**)   

**Figure 6:**
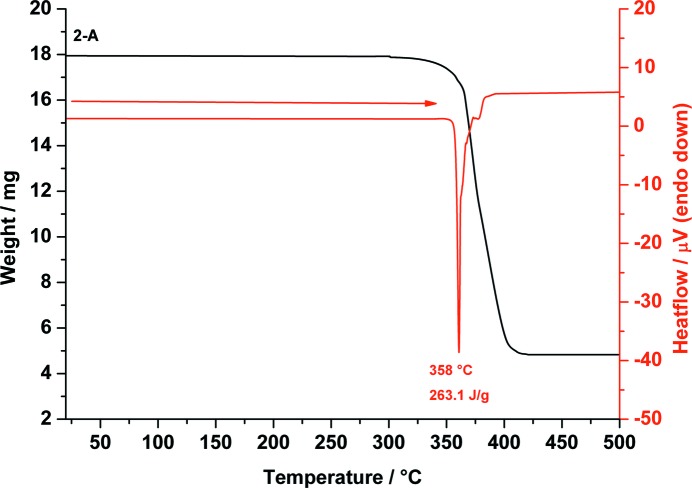
Combined DSC (red) and TGA (black) traces of *scyllo*-inositol (**2**-A) measured from 20 to 500 °C showing the decomposition of **2**-A at 358 °C.

DSC analysis of **2**-A shows only one sharp endothermic signal at 358 °C resulting from decomposition. TGA measurements show no mass loss or gain until 330 °C. Further heating results in decomposition (see Fig. 6 ▶).

To determine the unknown melting point of the second reported polymorph of *scyllo*-inositol (**2**-B, Yeon, 2001 ▶; Day *et al.*, 2006 ▶), a sample of pure **2**-B had to be prepared. Whereas samples of 100% **2**-A can be routinely obtained, **2**-B always crystallizes in the presence of **2**-A (Yeon, 2001 ▶; Day *et al.*, 2006 ▶). Repeated attempts to crystallize **2**-B using crystallization experiments from methanol/water as indicated in the publication of Day *et al.* failed to reproduce the polymorph. Vapour diffusion experiments were performed by dissolving 50, 40 and 30 mg samples of **2**-A in 3 ml water using an ultrasonic bath. The solutions were filtered using a filter paper with a porosity under 2.7 µm and filled into vials. The first set of solutions (containing 50, 40 and 30 mg dissolved in 3 ml water) were deposited without a lid into screw-top jars containing 10 ml methanol. In order to minimize the diffusion velocity of methanol into the solutions containing *scyllo*-inositol, the second set of vials was closed with snap-on lids perforated with a 0.9 mm cannula. Additionally, antisolvent crystallization experiments were performed by dissolving *scyllo*-inositol in the same manner as for the vapour diffusion experiments. Afterwards, portions of about 7 ml methanol were added, at first fast to each of the first set of experiments using a syringe and then slowly by placing methanol carefully over the solution containing *scyllo*-inositol to yield a two-phase system. In each experiment, different ratios of **2**-A and **2**-B were obtained, but these experiments also failed to produce pure **2**-B. We were therefore not able to determine the melting point of **2**-B. The DSC measurements of the mixtures of **2**-A and **2**-B showed two separate but barely resolved events, with onsets at about 359 and 364 °C.

The crystal structures of both polymorphs were reported by Yeon (2001 ▶); CSD reference codes EFURIH01 and EFURIH02 for **2**-A and **2**-B, respectively.

### 3.4. *neo*-Inositol (**3**) and *muco*-inositol (**4**)   

The crystal structures of *neo*-inositol (**3**) and *muco*-inositol (**4**) were reported by Yeon (2001 ▶; CSD reference code YEPNOW01) and Craig & James (1979 ▶; CSD reference code MUINOS), respectively. For their melting points, see Simperler *et al.* (2006 ▶). Considering the number of new phases discovered in our relatively straightforward heating experiments, it must be assumed that additional experiments on *neo*- and *muco*-inositol (not considered in our experiments) will reveal additional phases.

### 3.5. *cis*-Inositol (**5**)   

**Figure 7:**
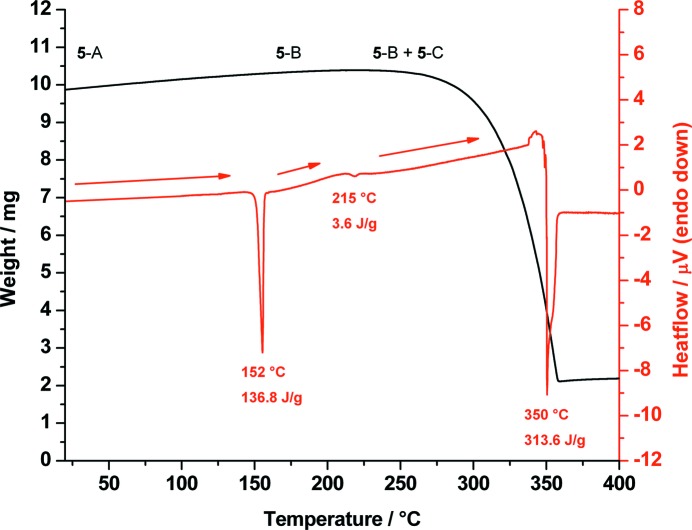
Combined DSC (red) and TGA (black) traces of *cis*-inositol (**5**-A) measured from 20 to 400 °C showing the phase transition of polymorph **5**-A to **5**-B at 152 °C and **5**-B to **5**-C at 215 °C until melting/decomposition of **5**-C at 350 °C.

DSC analysis of **5**-A shows a sharp endothermic signal at 152 °C resulting from the phase transition to a high-temperature form **5**-B. Furthermore, **5**-B shows a phase transition to another high-temperature form labelled as **5**-C. As was the case for d-**1**-B, the high value of the phase transition enthalpy from **5**-A to **5**-B is due to the fact that **5**-B and **5**-C are rotator phases. Upon further heating, a simultaneous melting/decomposition process occurs at 350 °C (Fig. 7 ▶).

**Figure 8:**
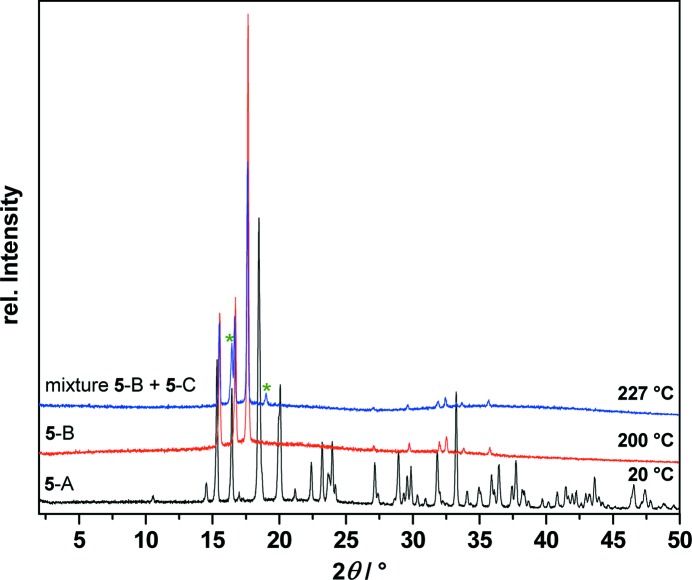
Temperature-dependent X-ray powder diffraction traces of *cis*-inositol (**5**) at 20, 200 and up to 227 °C showing the phase transition of polymorph **5**-A (black) to the first high-temperature polymorph **5**-B (red) to the second high-temperature polymorph **5**-C (blue). The asterisks (green) denote new reflections caused by polymorph **5**-C.

For identification of the polymorphs, T-XRPD patterns were measured before and after the phase transitions as shown in Fig. 8 ▶. The XRPD patterns show that the transition from **5**-B to **5**-C at 215 °C is incomplete, resulting in a mixture of **5**-B and **5**-C. However, the newly appearing peaks in **5**-C have a very different peak width (as measured by the full width at half maximum) than the peaks from **5**-B, which indicates that **5**-C is a true separate phase.

**Figure 9:**
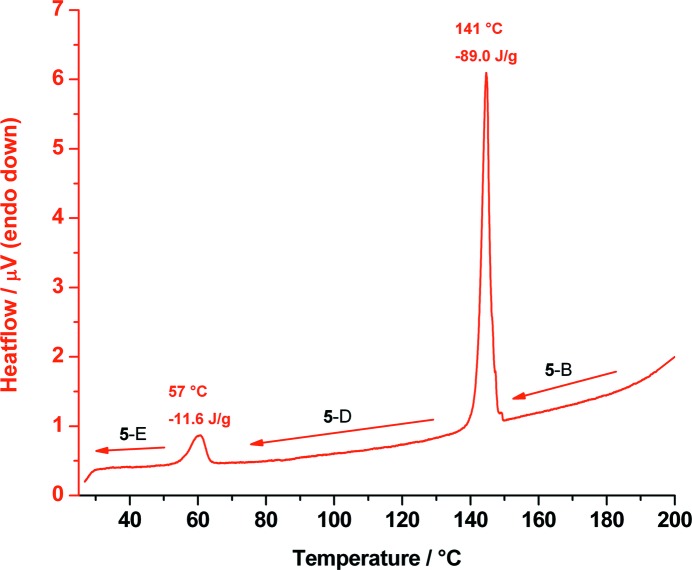
DSC trace of *cis*-inositol measured from 200 °C down to room temperature showing the phase transition of polymorph **5**-B to **5**-D at 141 °C and **5**-D to **5**-E at 57 °C.

**Figure 10:**
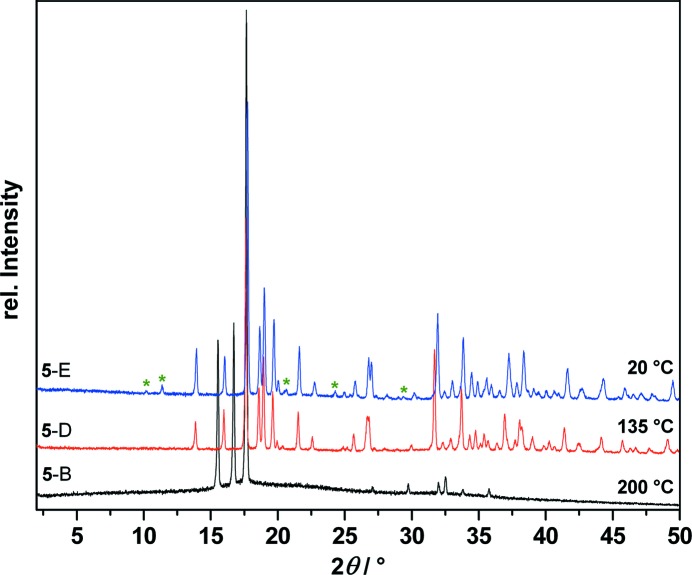
Temperature-dependent X-ray powder diffraction traces of *cis*-inositol (**5**) at 200, 135 and down to 20 °C showing the phase transitions of polymorph **5**-B (black) to polymorph **5**-D (red) to polymorph (**5**-E) (blue). **5**-D and **5**-E can be indexed with the same unit cell; the asterisks (green) denote the reflections that are visible in **5**-E but that are systematic absences in **5**-D.

When polymorph **5**-B is cooled from 200 °C to room temperature, it does not convert back to **5**-A, but forms two new polymorphs: at 141 °C form **5**-B transforms to **5**-D, which at 57 °C converts to form **5**-E (Fig. 9 ▶). Therefore, it can be assumed that **5**-D is an additional high-temperature form of *cis*-inositol. To identify the polymorphic forms that appeared during DSC measurement, T-XRPD patterns were recorded as shown in Fig. 10 ▶.

**Figure 11:**
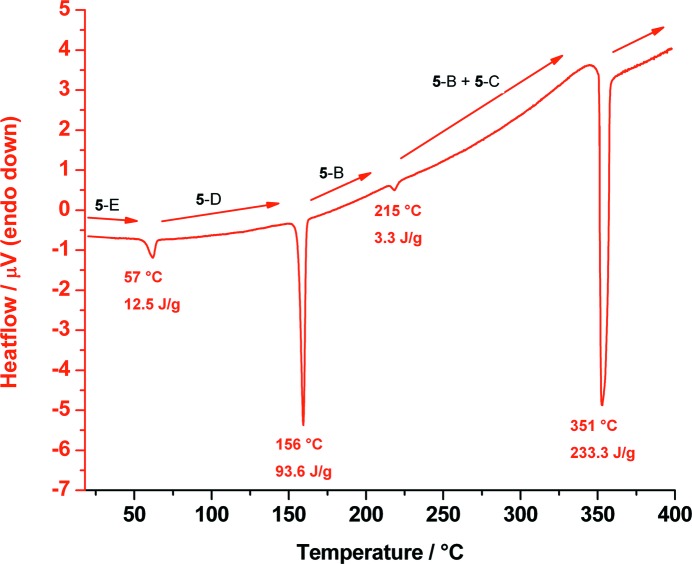
DSC trace of *cis*-inositol (**5**) measured from 20 up to 400 °C showing the phase transition of polymorph **5**-E back to **5**-D at 57 °C, **5**-D back to **5**-B at 156 °C and **5**-B to **5**-C at 215 °C until melting/decomposition of **5**-C at 351 °C.

**Figure 12:**
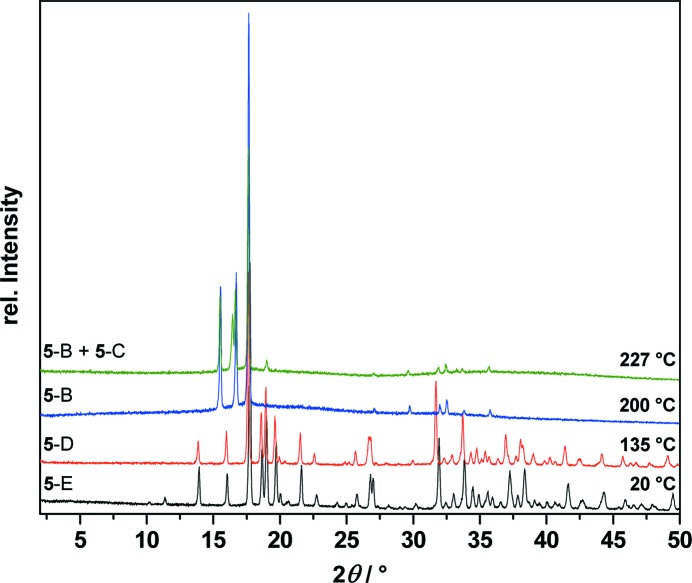
Temperature-dependent X-ray powder diffraction traces of *cis*-inositol (**5**) at 20, 135, 200 and up to 227 °C showing the phase transitions of **5**-E (black) to polymorph **5**-D (red) to polymorph **5**-B (blue) and finally to a mixture of polymorphs **5**-B and **5**-C (green).

These transformations are reversible: upon heating, **5**-E changes back to **5**-D at 57 °C, to **5**-B at 156 °C and to **5**-C at 215 °C, which finally shows a melting/decomposition point at 351 °C (Fig. 11 ▶). For the identification of the polymorphs occurring during the DSC measurement, T-XRPD patterns were measured before and after the phase transitions as shown in Fig. 12 ▶. After all T-XRPD measurements, a final rapid cooling process from 227 to 20 °C led to a conversion of polymorph **5**-C to **5**-E. The TGA curves show no mass loss or gain during these heating and cooling processes, except at the melting/decomposition points.

The crystal structures of the ordered phases **5**-A, **5**-D and **5**-E were solved and refined from laboratory X-ray powder diffraction data. The Rietveld plots are shown in the supporting information.

**Figure 13:**
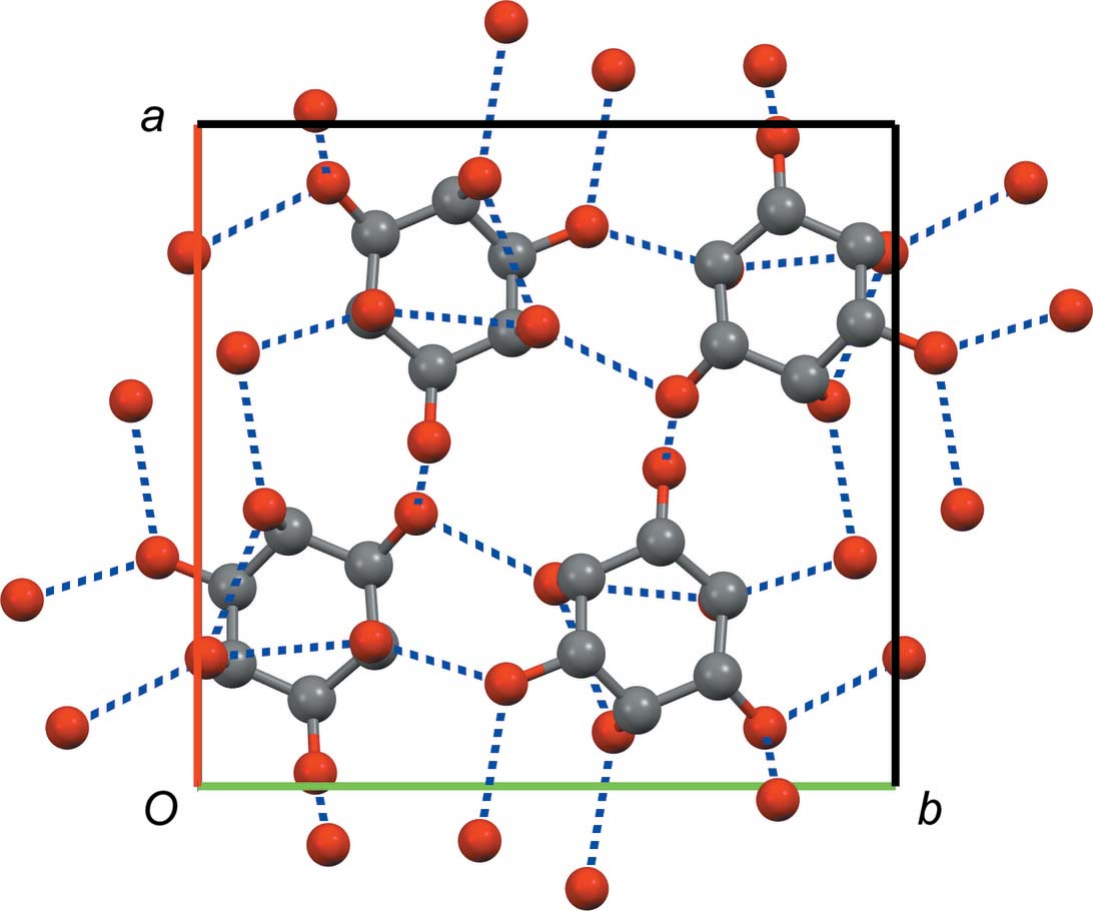
Crystal structure of **5**-A. Space group *P*2_1_/*n*, view along the *c* axis (*a* axis shown in red, *b* axis shown in green). Hydrogen bonds are indicated as dashed blue lines, H atoms have been omitted for clarity.

In **5**-A, each molecule forms one intramolecular hydrogen bond and ten intermolecular hydrogen bonds (five as donors, five as acceptors; Fig. 13 ▶).

**Figure 14:**
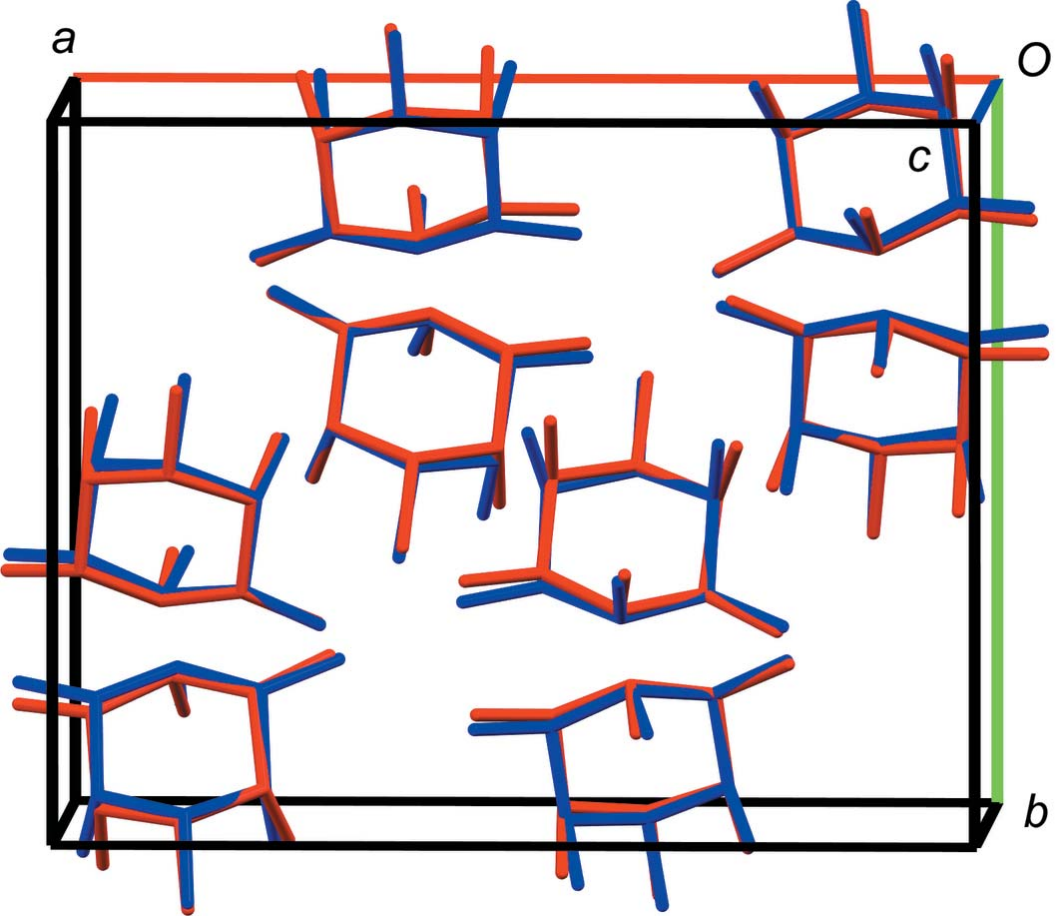
Overlay of the crystal structures of **5**-D (red, *Pbca*, *Z*′ = 1) and **5**-E (blue, *P*2_1_2_1_2_1_, *Z*′ = 2). View approximately along the *c* axis (*a* axis shown in red, *b* axis shown in green, *c* axis shown in blue), H atoms have been omitted for clarity.

**5**-D is a high-temperature polymorph that only exists above 57 °C and that converts to **5**-E on cooling. The crystal structures of **5**-D and **5**-E are very similar and share the same unit-cell parameters. The phase transition corresponds to the loss of the inversion symmetry to lower the space-group symmetry from *Pbca*, *Z*′ = 1 to one of its maximum subgroups *P*2_1_2_1_2_1_, *Z*′ = 2 (see overlay in Fig. 14 ▶). In **5**-D and **5**-E, each molecule forms one intramolecular and ten intermolecular hydrogen bonds.

Interestingly, the *C*_3*v*_-symmetrical *cis*-inositol (σ = 3) has five different polymorphs, of which two are rotator phases, the first even at quite a low temperature (156 °C). In contrast, the *D*_3*d*_-symmetrical *scyllo*-inositol (σ = 6) exhibits neither a rotator phase nor any other phase transition up to its decomposition at 355 °C.

The crystal structure of *cis*-inositol monohydrate (**5**·H_2_O) was determined by Freeman *et al.* (1996 ▶). This inositol phase is the only previously reported inositol phase with less than 12 hydrogen bonds per molecule. **5**·H_2_O crystallizes in *P*2_1_/*c* with two molecules in the asymmetric unit; one molecule forms 11 hydrogen bonds, the other only ten.

### 3.6. *allo*-Inositol (**6**)   

**Figure 15:**
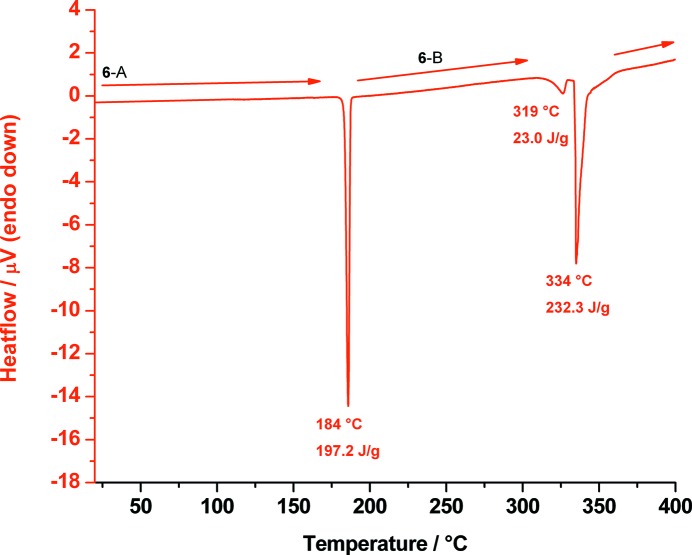
DSC trace of *allo*-inositol measured from 20 up to 400 °C showing the phase transition of polymorph **6**-A to **6**-B at 184 °C, melting of **6**-B at 319 °C and decomposition at 334 °C.

DSC analysis of *allo*-inositol shows a sharp endothermic signal with a minimum at about 184 °C resulting from the phase transition from polymorph **6**-A to the high-temperature polymorph **6**-B. Two further endothermic signals could be observed; the first onset at 319 °C resulting from melting of polymorph **6**-B and the second sharp endothermic signal at 334 °C resulting from decomposition. **6**-B is another rotator phase, again explaining the unusually high enthalpy of the transition from **6**-A to **6**-B (Fig. 15 ▶).

**Figure 16:**
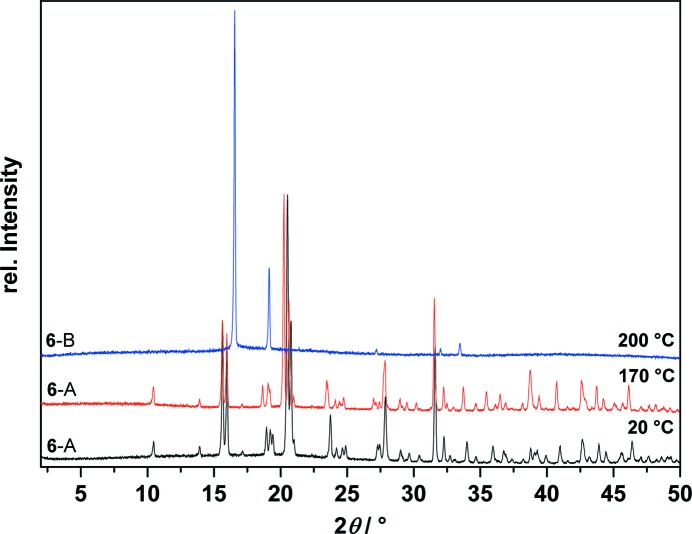
Temperature-dependent X-ray powder diffraction traces of *allo*-inositol (**6**) at 20, 170 and 200 °C showing the phase transition of polymorph **6**-A (black and red), which is stable up to the minimum 170 °C, to polymorph **6**-B at 200 °C (blue).

T-XRPD measurements were performed before and after the phase transition as observed in the DSC (Fig. 15 ▶), see Fig. 16 ▶.

The crystal structure of the room-temperature phase **6**-A was determined by Bonnet *et al.* (2006*a* ▶; CSD reference code IFAKAC); for the rotator phase **6**-B see §3.9.

### 3.7. *myo*-Inositol (**7**)   

**Figure 17:**
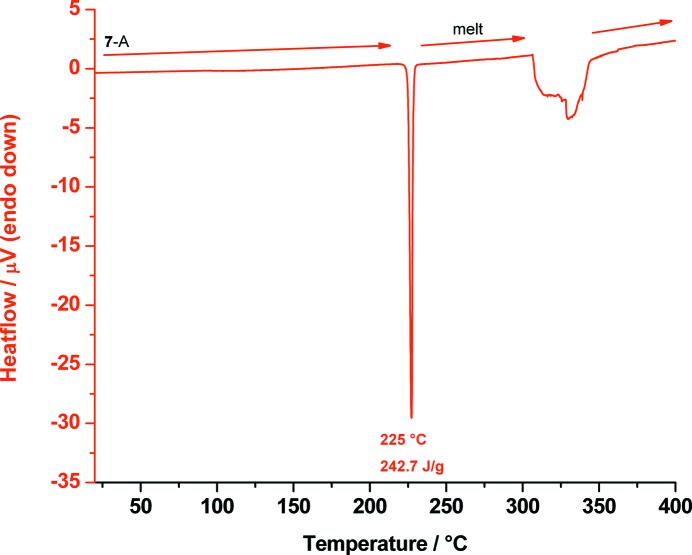
DSC trace of *myo*-inositol (**7**) measured from 20 up to 400 °C showing its melting point of polymorph **7**-A at 225 °C and its decomposition between 306 and 363 °C.

We redetermined the melting point of polymorph **7**-A using DSC measurement (Fig. 17 ▶). The crystal structure of **7**-A was published by Rabinovich & Kraut (1964 ▶; CSD reference code MYINOL).

To determine the unknown melting point of the second reported polymorph of *myo*-inositol (**7**-B, Khan *et al.*, 2007 ▶; CSD reference code MYINOL01), a sample of **7**-B had to be prepared. Repeated attempts to crystallize **7**-B including crystallizations from ethanol/ethyl acetate 60:40 as indicated in the publication of Khan *et al.* and additional solvent-assisted grinding experiments failed to reproduce the polymorph. The authors of the paper were contacted, but the sample was no longer available. We were therefore not able to determine the melting point of **7**-B.

**Figure 18:**
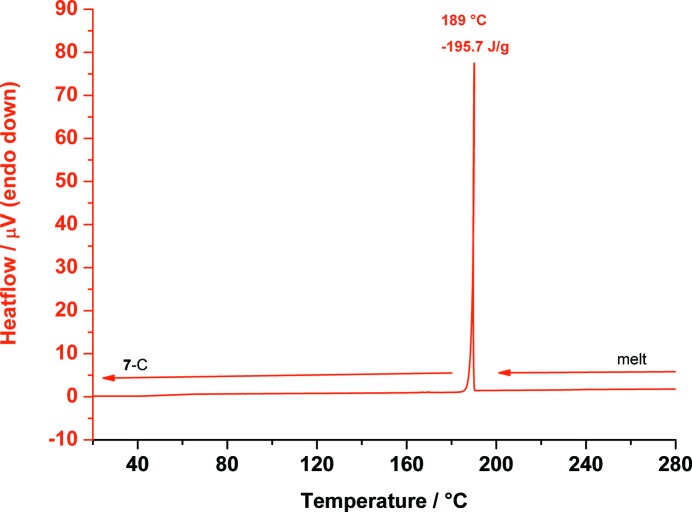
DSC trace of *myo*-inositol (**7**) measured from 280 down to 20 °C showing the transformation from the melt to polymorph **7**-C at 189 °C.

Although we did not obtain **7**-B, we could observe a third polymorph of *myo*-inositol (**7**-C) during thermal analyses on polymorph **7**-A. Polymorph **7**-C was obtained during DSC measurements by heating **7**-A to 280 °C until **7**-A had melted completely. During the cooling down process to 20 °C, **7**-C crystallizes from the melt at 189 °C and is stable at 20 °C (Fig. 18 ▶). It appears that a slow cooling rate yields form **7**-C from the melt, whereas a fast cooling rate yields form **7**-A from the melt.

**Figure 19:**
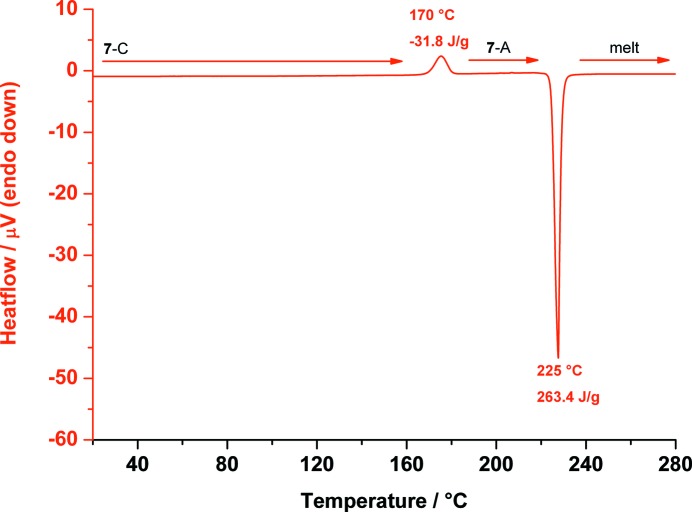
DSC trace of *myo*-inositol (**7**) measured from 20 up to 280 °C showing the phase transition of **7**-C back to **7**-A at 170 °C, and the melting point of polymorph **7**-A at 225 °C.

Heating **7**-C to 280 °C, at 170 °C it transforms back to **7**-A, which melts at 225 °C (see Fig. 19 ▶); this transition is reproducible.

**Figure 20:**
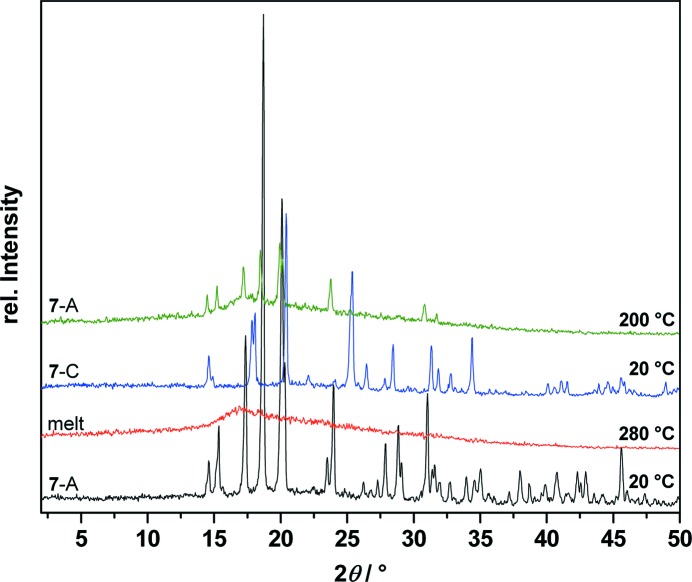
Temperature-dependent X-ray powder diffraction traces of *myo*-inositol (**7**) at 20 up to 280 down to 20 and up to 200 °C showing the melt of polymorph **7**-A (black and red), recrystallization to **7**-C (blue) and phase transition back to **7**-A (green).

T-XRPD measurements with the HUBER heater device and an imaging-plate position-sensitive detector were performed before and after the phase transitions observed in the DSC measurements (Fig. 20 ▶).

A final cool-down of the melt shown in Fig. 19 ▶ led to the recrystallization of polymorph **7**-A (see Fig. S13 ▶ in the supporting information).

At room temperature, **7**-C slowly converts to **7**-A over time. See the supporting information for further information.

The crystal structure of **7**-C was solved from laboratory X-ray powder diffraction data using real-space methods. The Rietveld refinement is shown in the supporting information.

**Figure 21:**
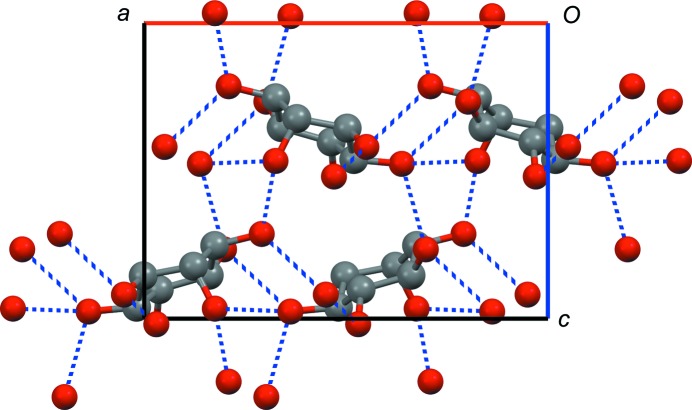
Crystal structure of **7**-C. Space group *Pca*2_1_, view along the *b* axis (*a* axis shown in red, *c* axis shown in blue). Hydrogen bonds are indicated as dashed blue lines, H atoms have been omitted for clarity.

The new polymorph of *myo*-inositol (**7**-C) crystallizes in *Pca*2_1_ with one molecule in the asymmetric unit. Each molecule is connected to the other molecules through 12 hydrogen bonds (Fig. 21 ▶).

### 3.8. *epi*-Inositol (**8**)   

The crystal structure of *epi*-inositol (**8**) was determined by Jeffrey & Kim (1971 ▶; CSD reference code EPINOS). For the melting point, see Simperler *et al.* (2006 ▶). Considering the number of new phases discovered in our relatively straightforward heating experiments, it must be assumed that additional experiments on *epi*-inositol, not considered in our experiments, will reveal additional phases.

### 3.9. Rotator phases   

The peak positions and intensities in the X-ray powder patterns of d-**1**-B, l-**1**-B, **5**-C and **6**-B are the same, and it must therefore be assumed that these phases – though consisting of chemically different molecules – are isostructural. The patterns contain only six peaks, which can be indexed with an orthorhombic, a tetragonal, a hexagonal or a cubic unit cell; these unit cells all have unit-cell parameters in common. Only the unit-cell volume of the cubic unit cell is chemically sensible, with the other unit-cell volumes being smaller than the volume of a single inositol molecule at room temperature. The volume of the cubic unit cell is 800 Å^3^ (*a* = 9.3 Å) and based on the systematic absences, it must be *F*-centred; this yields a plausible molecular volume of 200 Å^3^, which is about 8% larger than the molecular volume in the room-temperature phases. The Pawley refinements can be found in the supporting information.

We conclude from the unusually high space-group symmetry, the low densities, the high temperatures at which these phases occur and the high enthalpies for the transitions between the ordered phases to these high-temperature phases that these structures are rotator phases. That also explains how the crystal structures of three chemically different species can be isostructural.

The X-ray powder pattern of **5**-B consists of only nine reflections. The powder pattern could be indexed by a hexagonal cell without ambiguity (*a* = 6.575, *c* = 10.580 Å); the unit-cell volume is 396.05 Å^3^, corresponding to *Z* = 2. The Pawley refinement can be found in the supporting information.

As was the case for d-**1**-B, l-**1**-B, **5**-C and **6**-B, we conclude from the unusually high space-group symmetry, the low density, the high temperature at which this phase occurs and from the high transition energy between **5**-A and **5**-B, that **5**-B is also a rotator phase.

### 3.10. Calculation of corrected melting points   

Equation (4) in the paper by Wei (1999 ▶)
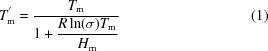
allows the calculation of corrected melting points: the melting point a compound would have if it had no internal symmetry. It is these corrected melting points that should be correlated with *e.g.* lattice energies, densities or number of hydrogen bonds. In equation (1), 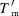 is the corrected melting point, *T*_m_ is the experimental melting point, *H*_m_ is the melting enthalpy and σ is the molecule’s symmetry number. Because of the observed polymorphism, it would be incorrect to speak of ‘the’ melting point for an inositol: each polymorph has its own *T*_m_, *H*_m_ and *T*_m_′, just like each polymorph has its own hydrogen-bonding pattern and lattice energy.

**Figure 22:**
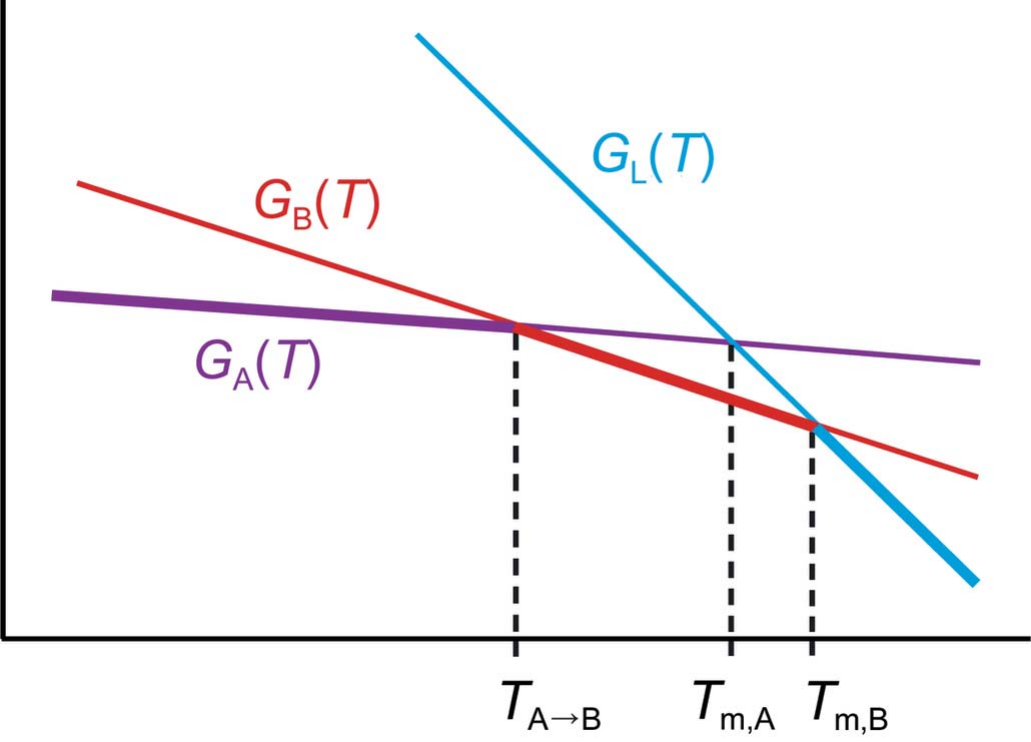
Virtual melting point *T*_m,A_ of phase A: the Gibbs free energies of phase A, phase B and the liquid as a function of temperature are shown. Phase A is the most stable phase at low temperature, and when the temperature increases phase A converts to phase B before melting. *T*_m,A_ and *H*_m,A_ cannot be measured directly (at ambient pressure), but *T*_m,A_ must lie between *T*_A→B_ and *T*_m,B_. The temperature dependence of the Gibbs free energies is represented as straight lines for clarity, in reality these lines are curved. A similar situation occurs when a phase decomposes before melting. The most stable phase at each temperature is shown in bold.

The quantitative evaluation of the corrected melting points through equation (1) is hampered by several problems:

(1) The definition of the molecular symmetry number σ in equation (1) assumes that the molecules are rigid. In our values for σ, we have ignored the flexible H atoms of the hydroxyl groups [a more rigorous calculation of σ for flexible molecules has been published (Gilson & Irikura, 2010 ▶), but this is beyond the scope of this paper].
(2) Many polymorphs show phase transitions below their melting point, in which case *T*_m_ and *H*_m_ cannot be measured directly (see Fig. 22 ▶). In principle, these values can be derived from other experimental data (Yu, 1995 ▶), but this has not been attempted in the current paper.
(3) *scyllo*-Inositol and *cis*-inositol decompose before melting.
(4) The correction that is applied is based on the assumption that the molecules in the liquid phase can rotate freely whereas those in the solid state do not rotate at all, causing the large difference in rotational entropy between the solid and the liquid phase. The rotator phases d-**1**-B, l-**1**-B, **5**-B, **5**-C and **6**-B, however, clearly violate this assumption.

Given these complications, we are not able to give a rigorous quantitative analysis of the melting points of the inositols. The only corrected melting point that can be calculated with the current data is that of *rac*-*chiro*-inositol (*rac*-**1**), for which *T*_m_′ = 221 °C.

## 4. Conclusions   

The aims of this work were to find the high-melting polymorph of *allo*-inositol (**6**-B), to determine *H*_m_ of *scyllo*-inositol (**2**-A), to determine the melting point of the second polymorph of *myo*-inositol (**7**-B) and to determine the crystal structures and corrected melting points of *rac*-*chiro*-inositol (*rac*-**1**) and *cis*-inositol (**5**).

We were able to identify the high-melting polymorph of *allo*-inositol (**6**-B) as a rotator phase, establish its unit cell and measure its melting point. *H*_A→B_ and *H*_m,B_ were also measured. *scyllo*-Inositol (**2**-A) decomposes before melting, and we were therefore not able to measure *H*_m_. The second known polymorph (**2**-B) could not be reproduced in pure form. The second polymorph of *myo*-inositol (**7**-B) proved elusive. A third polymorph was discovered (**7**-C), but it converts to the known first polymorph (**7**-A) before melting. Although *H*_m,A_ was measured, *myo*-inositol has no molecular symmetry and its melting point remains at 225 °C. We were able to solve the crystal structure of *rac*-*chiro*-inositol and to measure *H*_m_ and *T*_m_ to determine its corrected melting point as 221 °C. The phase behaviour of *cis*-inositol turned out to be unexpectedly complex. Five polymorphs were identified; for three of these (**5**-A, **5**-D and **5**-E), the crystal structures were solved from XRPD data, the remaining two structures are rotator phases (**5**-B and **5**-C). *cis*-Inositol decomposes before melting. Additionally, we established that the phase behaviour and crystal structures of l-*chiro*-inositol and d-*chiro*-inositol are the same, as expected.

Including hydrates and rotator phases, and counting enantiomers separately, 13 new phases are reported in this paper, bringing the total number of known phases for the inositols to 24, of which four are hydrates and five are rotator phases.

Our experiments have revealed a complex picture of phases, rotator phases and phase transitions, in which a simple correlation between melting points and hydrogen-bonding patterns is not feasible. A thorough discussion of the melting points of these 24 phases requires future work to determine the virtual melting points.

CCDC deposition numbers: 891302–891305, 891307 and 891309.

## Supplementary Material

Crystal structure: contains datablock(s) global, 5-A, 5-D, 5-E, 7-C, D-1-A, rac-1. DOI: 10.1107/S2052252513026511/bi5002sup1.cif

Rietveld powder data: contains datablock(s) 5-A. DOI: 10.1107/S2052252513026511/bi50025-Asup2.rtv

Rietveld powder data: contains datablock(s) 5-D. DOI: 10.1107/S2052252513026511/bi50025-Dsup3.rtv

Rietveld powder data: contains datablock(s) 5-E. DOI: 10.1107/S2052252513026511/bi50025-Esup4.rtv

Rietveld powder data: contains datablock(s) 7-C. DOI: 10.1107/S2052252513026511/bi50027-Csup5.rtv

Rietveld powder data: contains datablock(s) D-1-A. DOI: 10.1107/S2052252513026511/bi5002D-1-Asup6.rtv

Rietveld powder data: contains datablock(s) rac-1. DOI: 10.1107/S2052252513026511/bi5002rac-1sup7.rtv

Electronic Supplementary Material.. DOI: 10.1107/S2052252513026511/bi5002sup8.pdf

CCDC references: 891302, 891303, 891304, 891305, 891307, 891309

## Figures and Tables

**Table 1:** Overview of the polymorphs (not including hydrates) of the inositols and their phase transition temperatures

Isomer	Phase	*ρ* (g cm^−3^)	Space group	*T*_m_ (°C)	Δ*H*_t_ (J g^−1^)	Type of phase transition	Reference
D-(+)-*chiro*	D-**1**-A	1.60	*P*2_1_	201	181.9	Conversion to **1**-B	This work
D-(+)-*chiro*	D-**1**-B	1.50	F***†	245‡	17.0	Melting	This work
L-(−)-*chiro*	L-**1**-A	1.60	*P*2_1_	202	191.0	Conversion to **1**-B	Jeffrey & Yeon (1987 ▶)
L-(−)-*chiro*	L-**1**-B	1.50	F***†	246‡	16.4	Melting	This work
racemic	*rac*-**1**	1.69	*P*2_1_/*c*	250‡	243.1	Melting	This work
*scyllo*	**2**-A	1.57	*P*2_1_/*c*	358§	263.1	Decomposition	Yeon (2001 ▶), Day *et al.* (2006 ▶)
*scyllo*	**2**-B	1.66	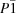	360§	–	–	Yeon (2001 ▶), Day *et al.* (2006 ▶)
*neo*	**3**	1.70	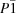	315¶	–	Melting	Yeon (2001 ▶)
*muco*	**4**	1.65	*P*2_1_/*c*	290¶	–	Melting	Craig & James (1979 ▶)
*cis*	**5**-A	1.61	*P*2_1_/*n*	152	136.8	Conversion to **5**-B	This work
*cis*	**5**-B	1.51	*P*3**/*P*6**††	215	> 3.6‡‡	Conversion to **5**-C	This work
*cis*	**5**-C	1.47	F*** †	351	313.6	Decomposition	This work
*cis*	**5**-D	1.63	*Pbca*	156	93.6	Conversion to **5**-B	This work
*cis*	**5**-E	1.66	*P*2_1_2_1_2_1_	57	12.5	Conversion to **5**-D	This work
*allo*	**6**-A	1.68	*P*2_1_/*n*	184	197.2	Conversion to **6**-B	Bonnet *et al.* (2006*a* ▶)
*allo*	**6**-B	1.50	F***†	319‡	23.04	Melting	This work
*myo*	**7**-A	1.58	*P*2_1_/*c*	225‡	242.7	Melting	Rabinovich & Kraut (1964 ▶)
*myo*	**7**-B	1.65	*Pna*2_1_	–	–	–	Khan *et al.* (2007 ▶)
*myo*	**7**-C	1.66	*Pca*2_1_	170	−31.8	Conversion to **7**-A	This work
*epi*	**8**	1.66	*P*2_1_/*c*	304¶	–	Melting	Jeffrey & Kim (1971 ▶)

† Rotator phase, cubic, space group unknown, see text.

‡ Onset/offset melting point from DSC measurements in this publication.

§ Melting points of **2**-A and **2**-B given as 360 °C by Yeon (2001 ▶); we observed decomposition at 358 °C for **2**-A.

¶ See Simperler *et al.* (2006 ▶).

†† Rotator phase, hexagonal, space group unknown, see text.

‡‡ Conversion is incomplete.

**Table 2:** Crystallographic data for the structures determined from X-ray powder diffraction data

	*rac*-**1**	D-**1**-A	**5**-A	**5**-D	**5**-E	**7**-C
Crystal data						
Chemical formula	C_6_H_12_O_6_	C_6_H_12_O_6_	C_6_H_12_O_6_	C_6_H_12_O_6_	C_6_H_12_O_6_	C_6_H_12_O_6_
*M*_r_	180.16	180.16	180.16	180.16	180.16	180.16
Crystal system, space group	Monoclinic, *P*2_1_/*c*	Monoclinic, *P*2_1_	Monoclinic, *P*2_1_/*n*	Orthorhombic, *Pbca*	Orthorhombic, *P*2_1_2_1_2_1_	Orthorhombic, *Pca*2_1_
Temperature (K)	293	293	293	408	293	293
*a* (Å)	10.1435 (6)	6.86637 (11)	11.58792 (19)	14.1313 (2)	14.01476 (14)	11.8577 (3)
*b* (Å)	8.1542 (4)	9.12272 (14)	12.2101 (2)	11.0757 (2)	11.03782 (11)	7.01486 (16)
*c* (Å)	8.6239 (4)	6.21914 (10)	5.25364 (10)	9.36191 (18)	9.33193 (12)	8.68032 (19)
*α* (°)	90	90	90	90	90	90
*β* (°)	92.3556 (15)	106.5963 (6)	90.5649 (7)	90	90	90
*γ* (°)	90	90	90	90	90	90
*V* (Å^3^)	712.70 (7)	373.338 (10)	743.30 (2)	1465.27 (5)	1443.58 (3)	722.03 (3)
*V*_mol_ (Å^3^)	178	187	186	183	180	181
*Z*	4	2	4	8	8	4
Radiation type	Cu *K*α_1_, λ = 1.54056 Å					
μ (mm^−1^)	1.33	1.27	1.28	1.30	1.31	1.31
Specimen shape, size (mm)	Cylinder, 10 × 0.7	Cylinder, 10 × 0.7	Cylinder, 10 × 0.7	Cylinder, 10 × 0.7	Cylinder, 10 × 0.7	Cylinder, 10 × 0.7
						
Data collection						
Diffractometer	Stoe Stadi-P diffractometer					
Specimen mounting	Glass capillary					
Data collection mode	Transmission					
Scan method	Step					
2θ values (°)	2θ_min_ = 2.0, 2θ_max_ = 79.99, 2θ_step_ = 0.01					
						
Refinement†						
*R*_wp_	0.0577	0.0329	0.0408	0.0356	0.0331	0.04336
*R*_p_	0.0411	0.0252	0.0304	0.0259	0.0244	0.0326
*R*_exp_	0.0198	0.0239	0.0302	0.0274	0.0274	0.0369
*R*_wp_′	0.1235	0.0787	0.0827	0.0816	0.0686	0.1021
*R*_p_′	0.1024	0.0793	0.0779	0.0758	0.0611	0.1117
*R*_exp_′	0.0423	0.0571	0.0613	0.0628	0.0567	0.0870
χ^2^	8.510	1.896	1.825	1.687	1.464	1.378
No. of data points	7800	7800	7800	7800	7800	7599
No. of parameters	102	69	64	65	90	65
No. of restraints	66	66	66	66	132	66
H-atom treatment	Calculated ‡	Calculated ‡	Calculated ‡	Calculated ‡	Calculated ‡	Calculated ‡

† *R*′_wp_, *R*′_p_ and *R*′_exp_ denote the values after background subtraction.

‡ Calculated by molecular dynamics followed by energy-minimization with DFT-D (see text).
