# Supplementary material for: On the correlation between hydrogen bonding and melting points in the inositols
Source: IUCrJ. 2013 Oct 18;1(Pt 1):61–73. doi: 10.1107/S2052252513026511 (PMC4104965; doi:10.1107/S2052252513026511)
Supplement: Supplementary file 8 [file m-01-00061-sup8.pdf]

IUCrJ

Volume 1 (2014)

Supporting information for article:

On the correlation between hydrogen bonding and melting points in the inositols

Sándor L. Bekö, Edith Alig, Martin U. Schmidt and Jacco van de Streek

Bekö, S. L. , Alig, E. , Schmidt, M. U. & Van de Streek, J. (2014). **IUCrJ**, **1**
doi:10.1107/S2052252513026511

Electronic Supporting Information

S1. Elemental analysis

Calculated for $C_6H_{12}O_6$ (%): C, 40.00; H, 6.71.
Found for D-**1**-A: C, 39.74; H, 6.37.
Found for *rac*-**1**: C, 39.70; H, 6.72.
Found for **2**-A: C, 40.08; H, 6.80.
Found for **5**-E: C, 39.76; H, 6.58.
Found for **7**-C: C, 39.92; H, 6.61.

S2. Rietveld refinements

The isostructurality (bar a reflection) of the crystal structure of D-(+)-chiro-inositol with that of L-(−)-chiro-inositol was confirmed through comparison of the experimental powder diffraction pattern of D-**1**-A with the powder pattern simulated from the single crystal structure of L-**1**-A. A crystal structure for D-**1**-A was obtained by starting from the single crystal structure of L-**1**-A and multiplying all atomic coordinates by -1. The resulting crystal structure was Rietveld refined with *TOPAS-Academic* 4.1. The positions of the hydrogen atoms were energy-optimised with a dispersion-corrected density functional theory method keeping the positions of the non-hydrogen atoms and the unit cell fixed.

The Rietveld plot for D-**1**-A is given in Fig. S1. The cif file for the crystal structure is part of the ESI of this paper.

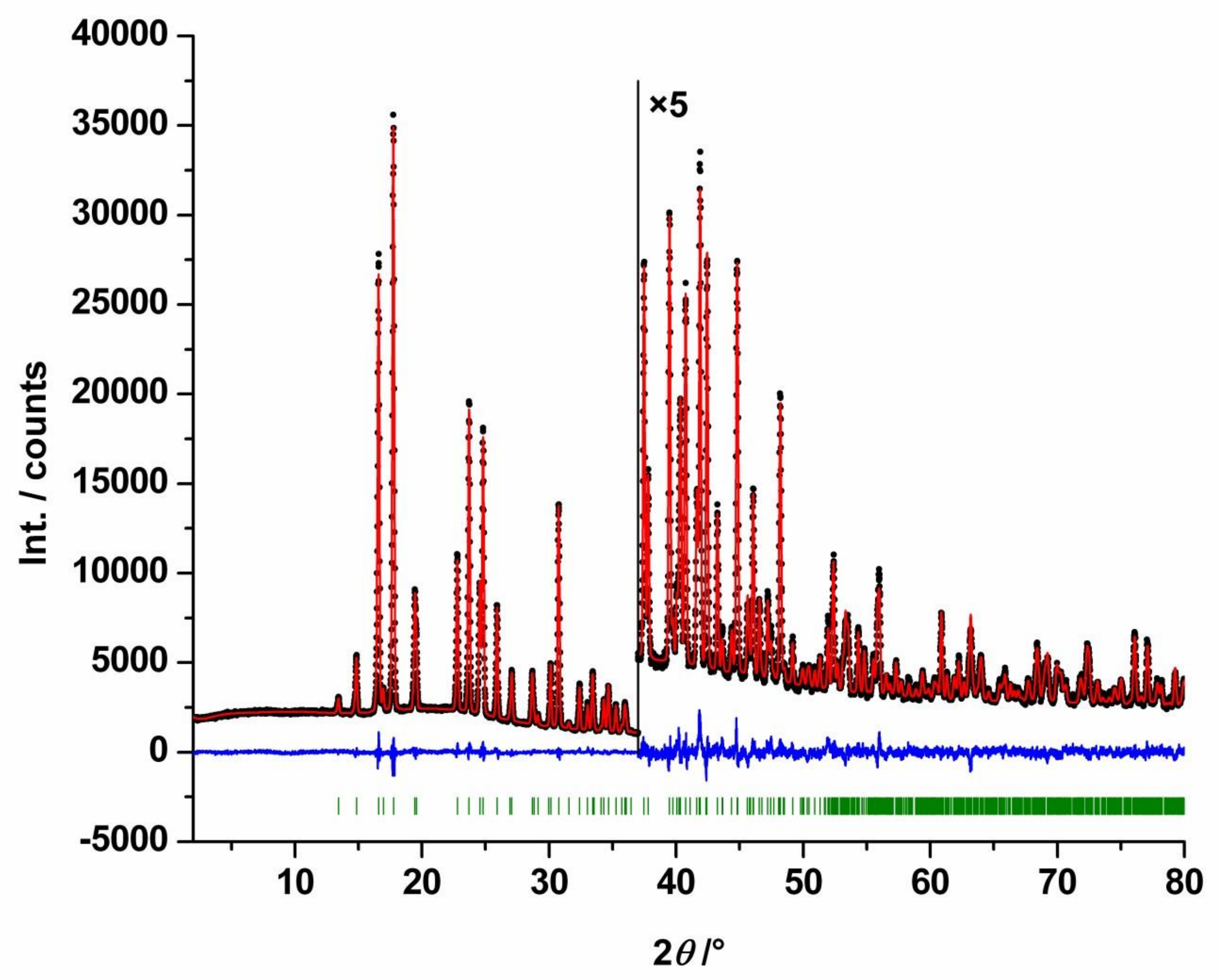

Figure S1 Rietveld plot of D-**1**-A: observed (black), calculated (red) and difference (blue) profiles and tick marks (green). At about 36° in 2θ the scale changes by a factor of 5.

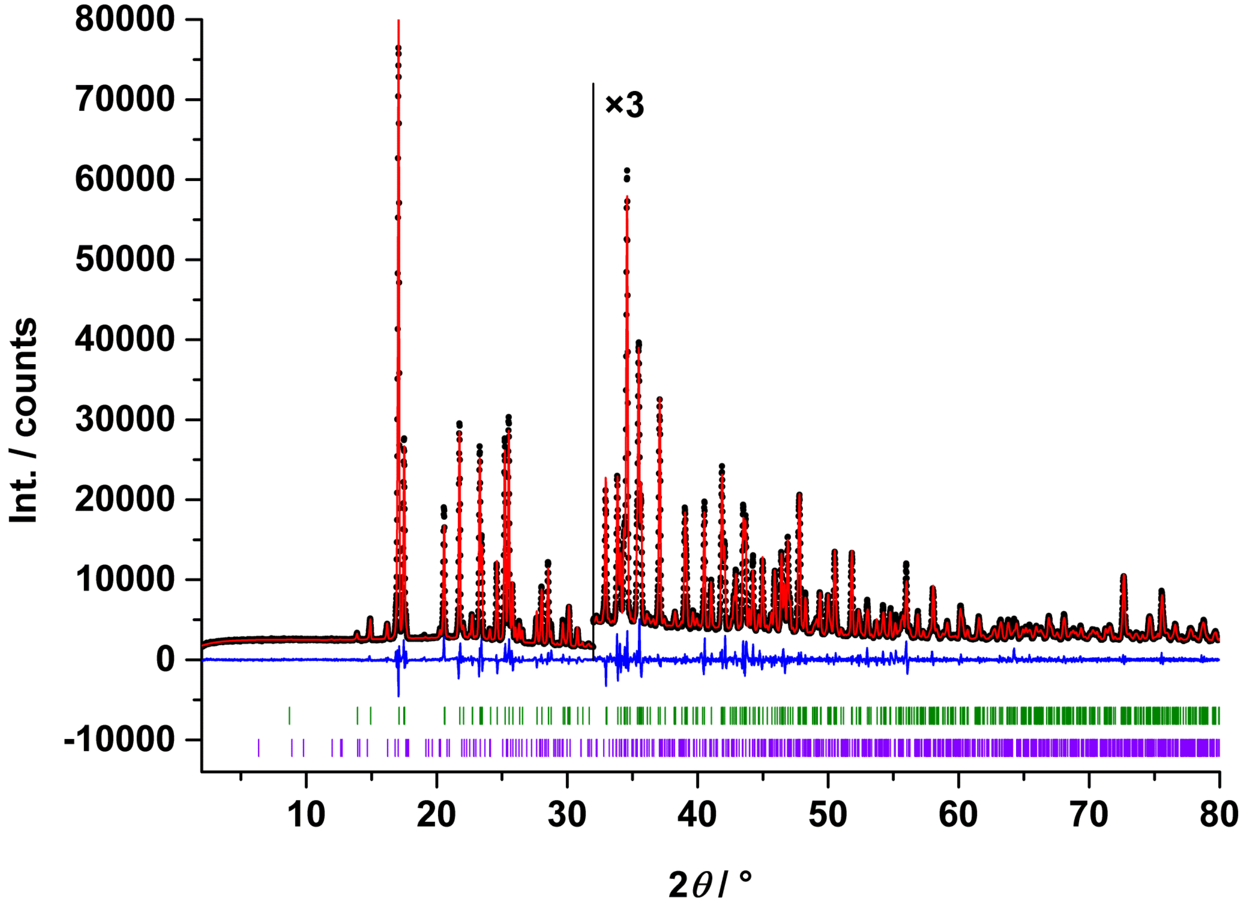

Figure S2 Rietveld plot of *rac*-**1**: observed (black), calculated (red) and difference (blue) profiles and tick marks for *rac*-**1** (green) and D/L-**1**·⅓H_2O (magenta). At about 32° in 2*θ* the scale changes by a factor of 3.

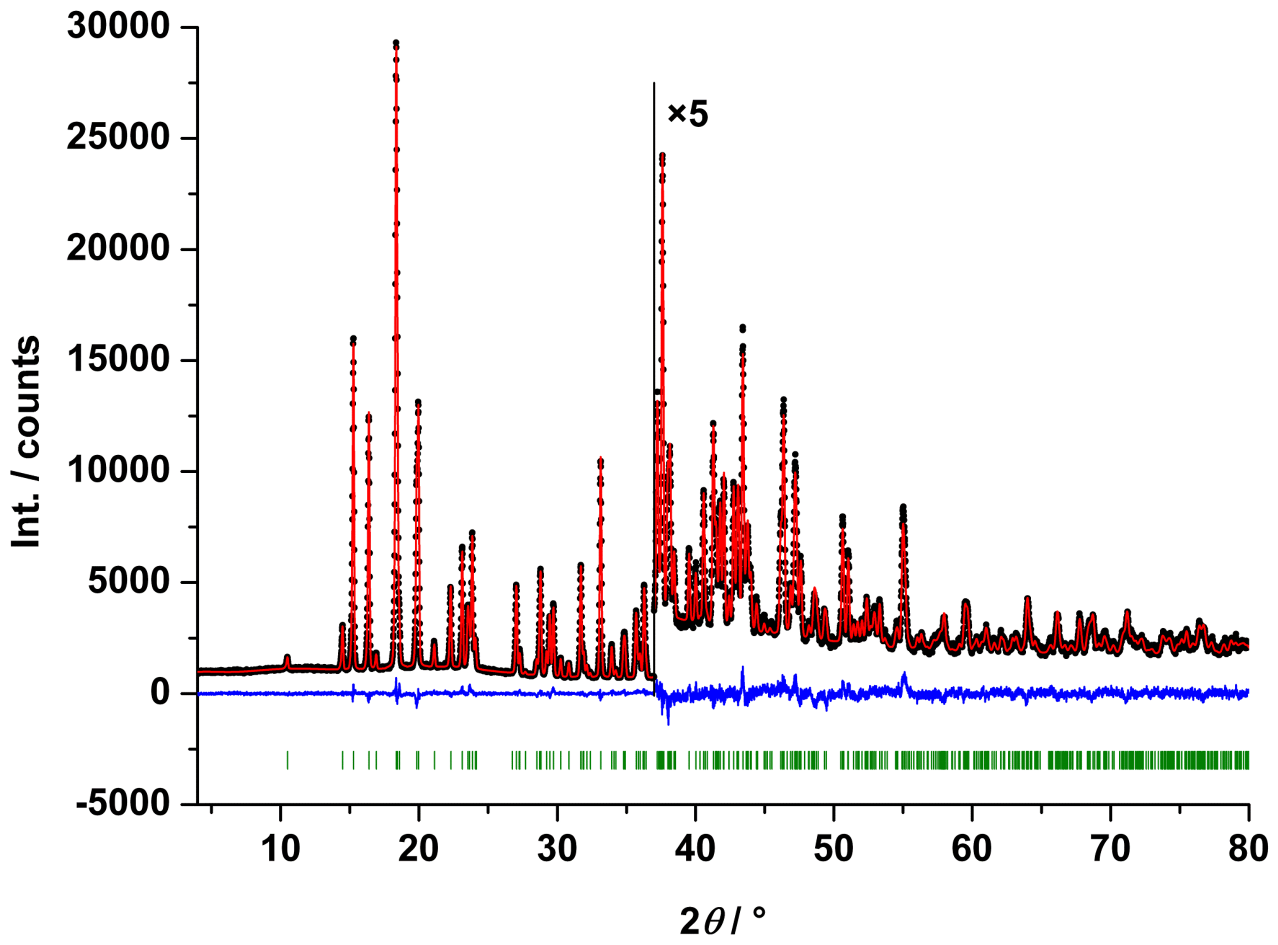

Figure S3 Rietveld plot of **5**-A: observed (black), calculated (red) and difference (blue) profiles and tick marks (green). At about 36° in 2*θ* the scale changes by a factor of 5.

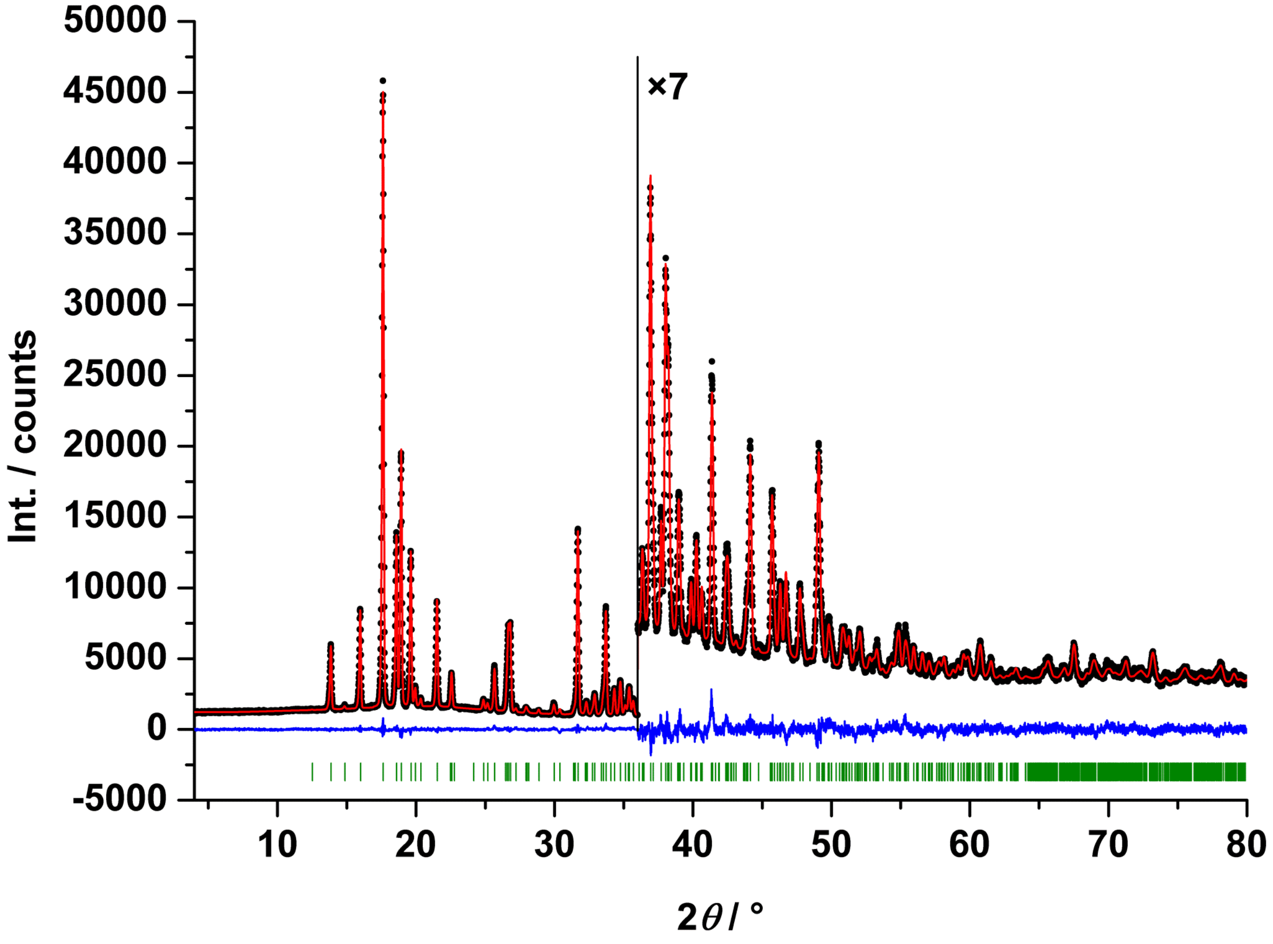

Figure S4 Rietveld plot of **5**-D: observed (black), calculated (red) and difference (blue) profiles and tick marks (green). At about 36° in 2θ the scale changes by a factor of 7.

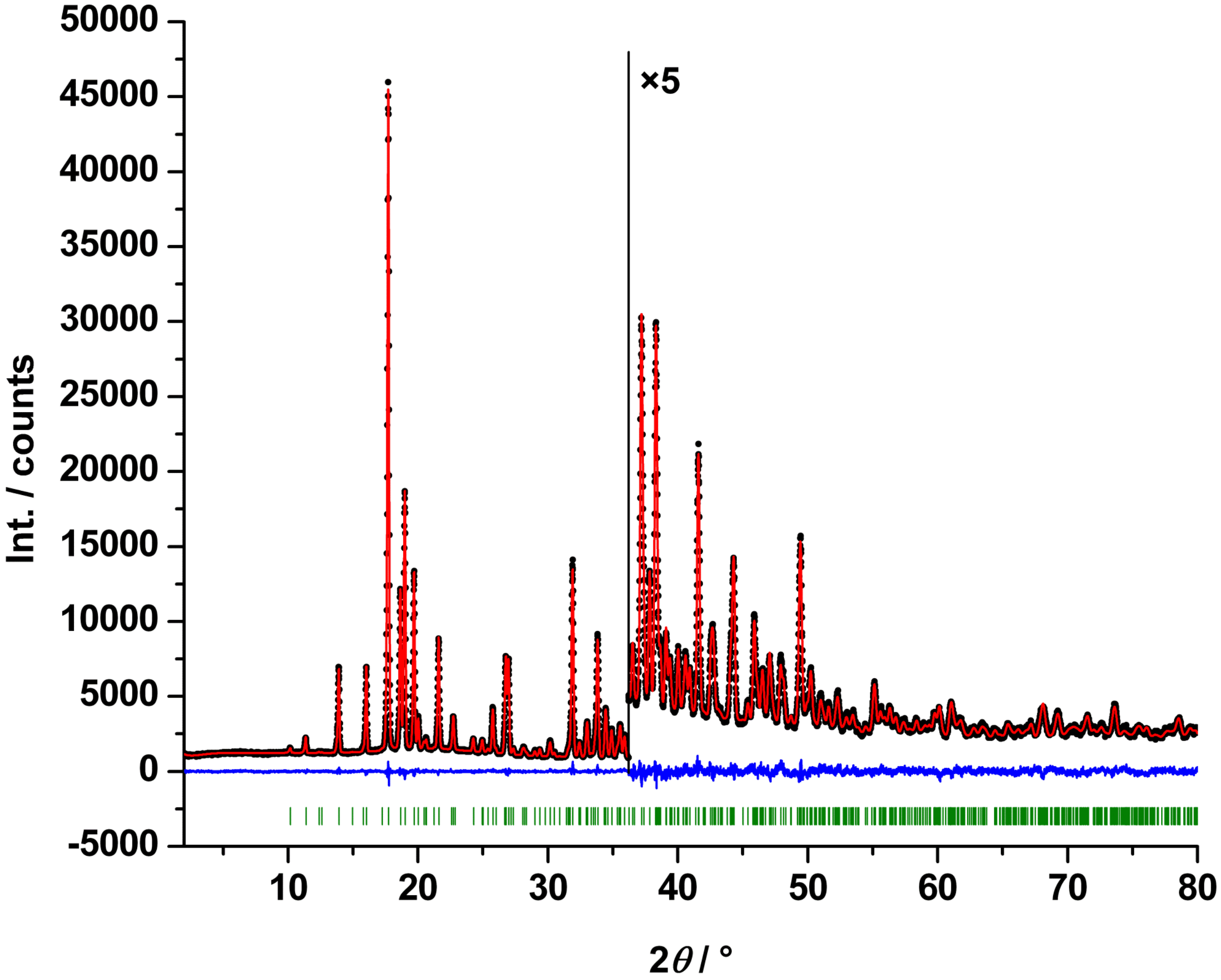

Figure S5 Rietveld plot of **5**-E: observed (black), calculated (red) and difference (blue) profiles and tick marks (green). At about 36° in 2θ the scale changes by a factor of 5.

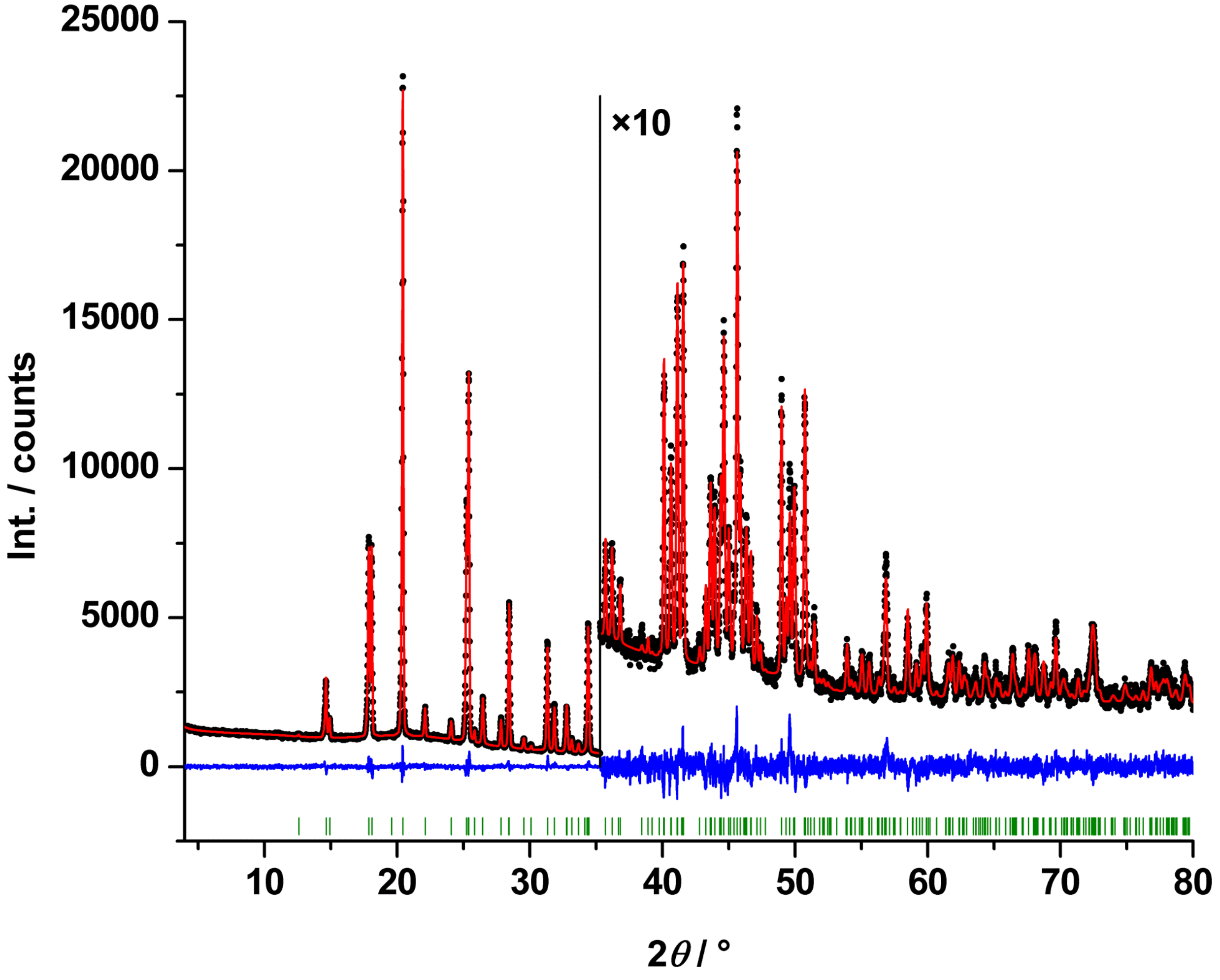

Figure S6 Rietveld plot of **7**-C: observed (black), calculated (red) and difference (blue) profiles and tick marks (green). At about 36° in 2*θ* the scale changes by a factor of 10.

S3. Pawley refinements on the rotator phases L-1-B, D-1-B, 5-C, 6-B and 5-B

All phases were measured with Cu-$K_{\alpha 1}$ radiation (λ = 1.5406 Å) in the range 2θ = 2-80° (the mixture of **5**-C and **5**-B was measured in the range 2-60°). All phases have chemical formula $C_6H_{12}O_6$, M_r = 180.16 g/mol. The Pawley fits, carried out with *TOPAS* 4.1, converged well for D-**1**-B:, *F**, *a* = 9.31568 Å, *V* = 808.432 $Å^3$, *Z* = 4, measurement at 500 K, R_{wp} = 0.0304, R_p = 0.0233, R_{exp} = 0.0280 (before background subtraction), R'_{wp} = 0.1584, R'_p = 0.1925, R'_{exp} = 0.1460 (after background subtraction), χ^2 = 1.085, for L-**1**-B: *F**, *a* = 9.31210 Å, *V* = 807.501 $Å^3$, *Z* = 4, measurement at 500 K, R_{wp} = 0.0254, R_p = 0.0190, R_{exp} = 0.0190 (before background subtraction), R'_{wp} = 0.2013, R'_p = 0.2191, R'_{exp} = 0.1506 (after background subtraction), χ^2 = 1.786, for **5**-C: *F**, *a* = 9.33440 Å, *V* = 813.316 $Å^3$, *Z* = 4, measurement at 500 K, R_{wp} = 0.0601, R_p = 0.0461, R_{exp} = 0.0635 (before background subtraction), R'_{wp} = 0.2018, R'_p = 0.2947, R'_{exp} = 0.2132 (after background subtraction), χ^2 = 0.947 and for **6**-B: *F**, *a* = 9.26836 Å, *V* = 796.175 $Å^3$, *Z* = 4, measurement at 473 K, R_{wp} = 0.0404, R_p = 0.0304, R_{exp} = 0.0341 (before background subtraction), R'_{wp} = 0.1925, R'_p = 0.2689, R'_{exp} = 0.1624 (after background subtraction), χ^2 = 1.185. For **5**-B: *P*3*/*P*6*, *a* = 6.57509 Å, *c* = 10.5818 Å, *V* = 396.181 $Å^3$, *Z* = 2, measurement at 473 K, R_{wp} =

0.0296, R_p = 0.0225, R_{exp} = 0.0263 (before background subtraction), R'_{wp} = 0.1369, R'_p = 0.1663, R'_{exp} = 0.1214 (after background subtraction), χ^2 = 1.127.

The Pawley plots for D-**1**-B, L-**1**-B, **5**-C, **6**-B and **5**-B are given in Fig. S7, Fig. S8, Fig. S9, Fig. S10 and Fig. S11, respectively.

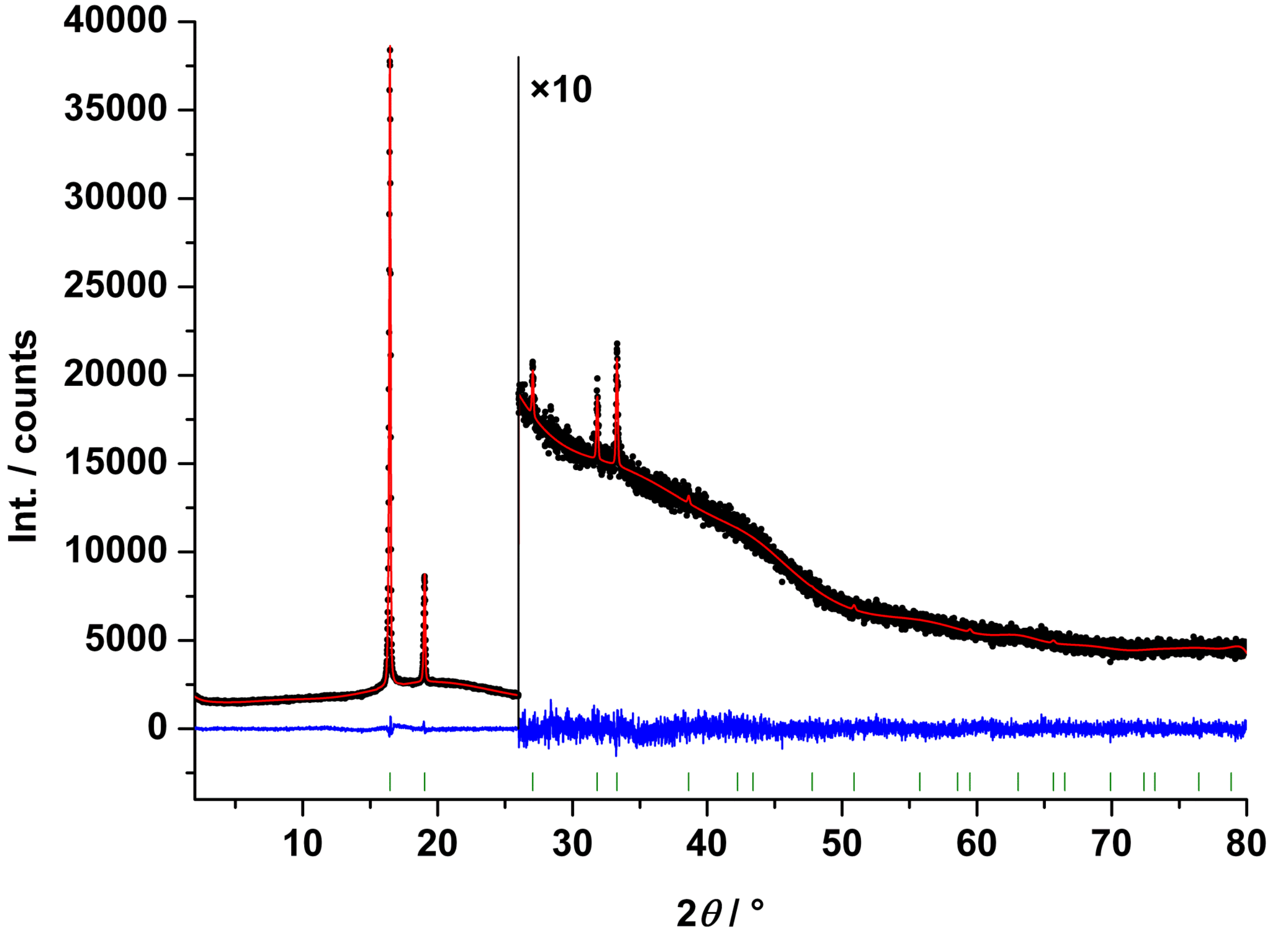

Figure S7 Pawley plot of D-**1**-B: observed (black), calculated (red) and difference (blue) profiles and tick marks (green).

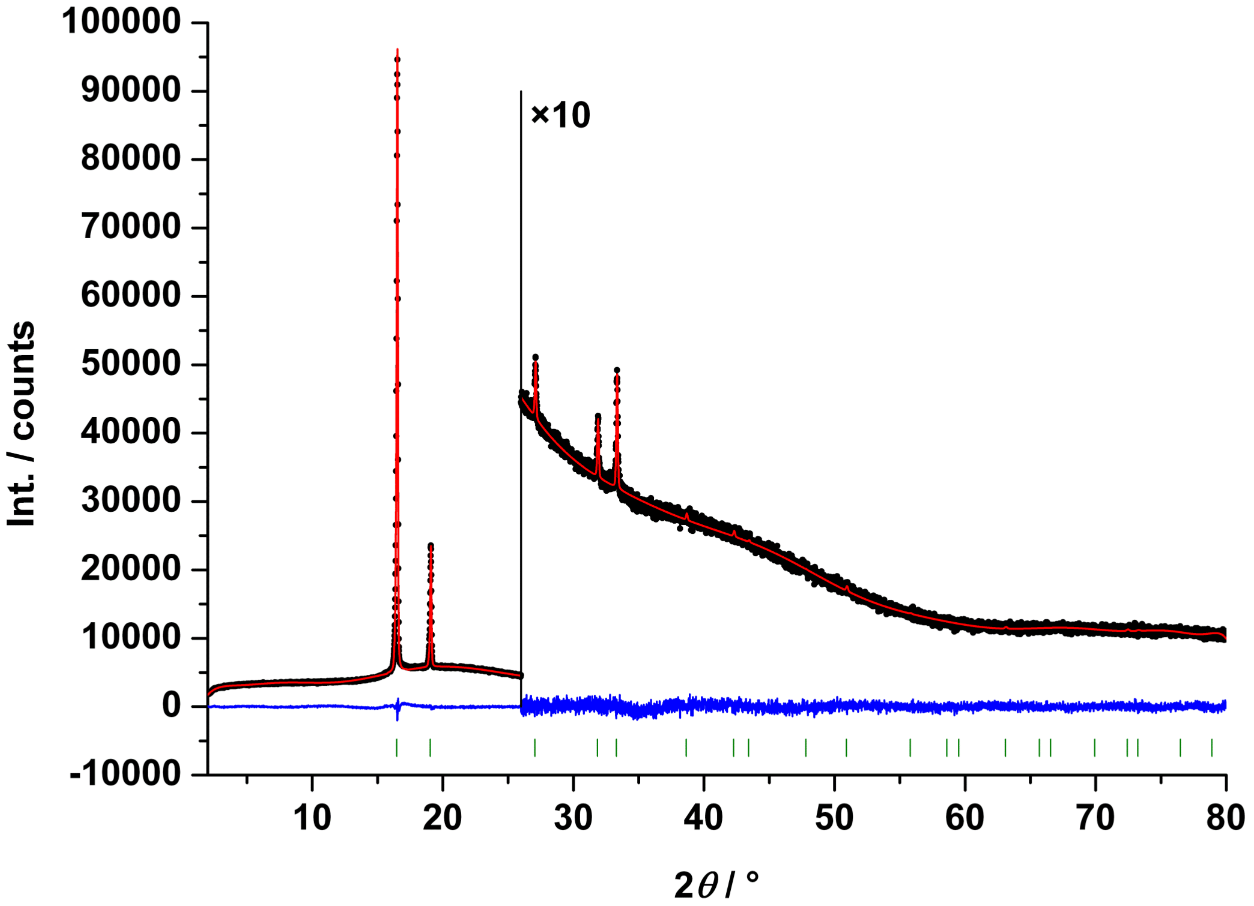

Figure S8 Pawley plot of L-**1**-B: observed (black), calculated (red) and difference (blue) profiles and tick marks (green).

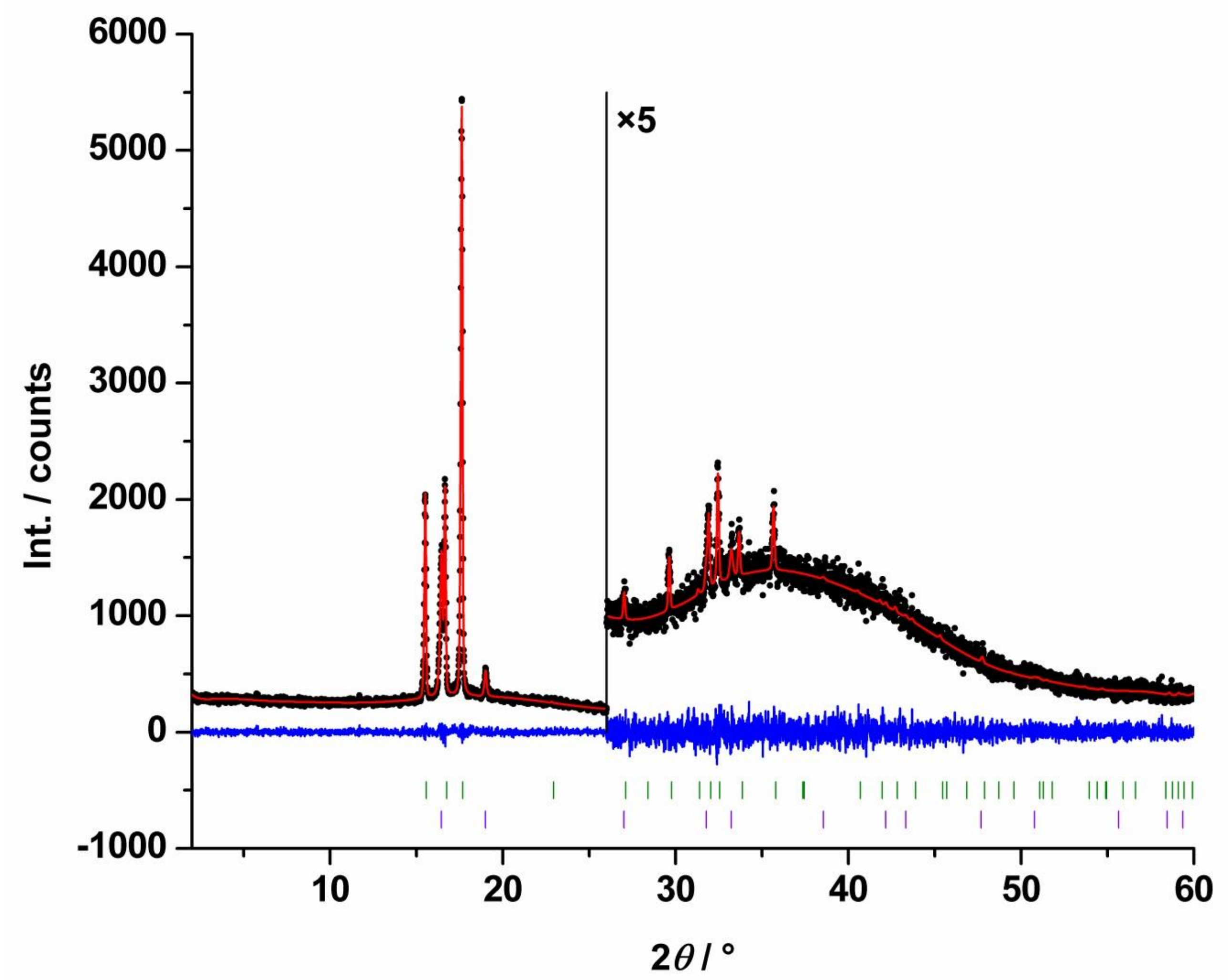

Figure S9 Pawley plot of **5**-C with **5**-B present: observed (black), calculated (red) and difference (blue) profiles and tick marks for **5**-B (green) and **5**-C (magenta).

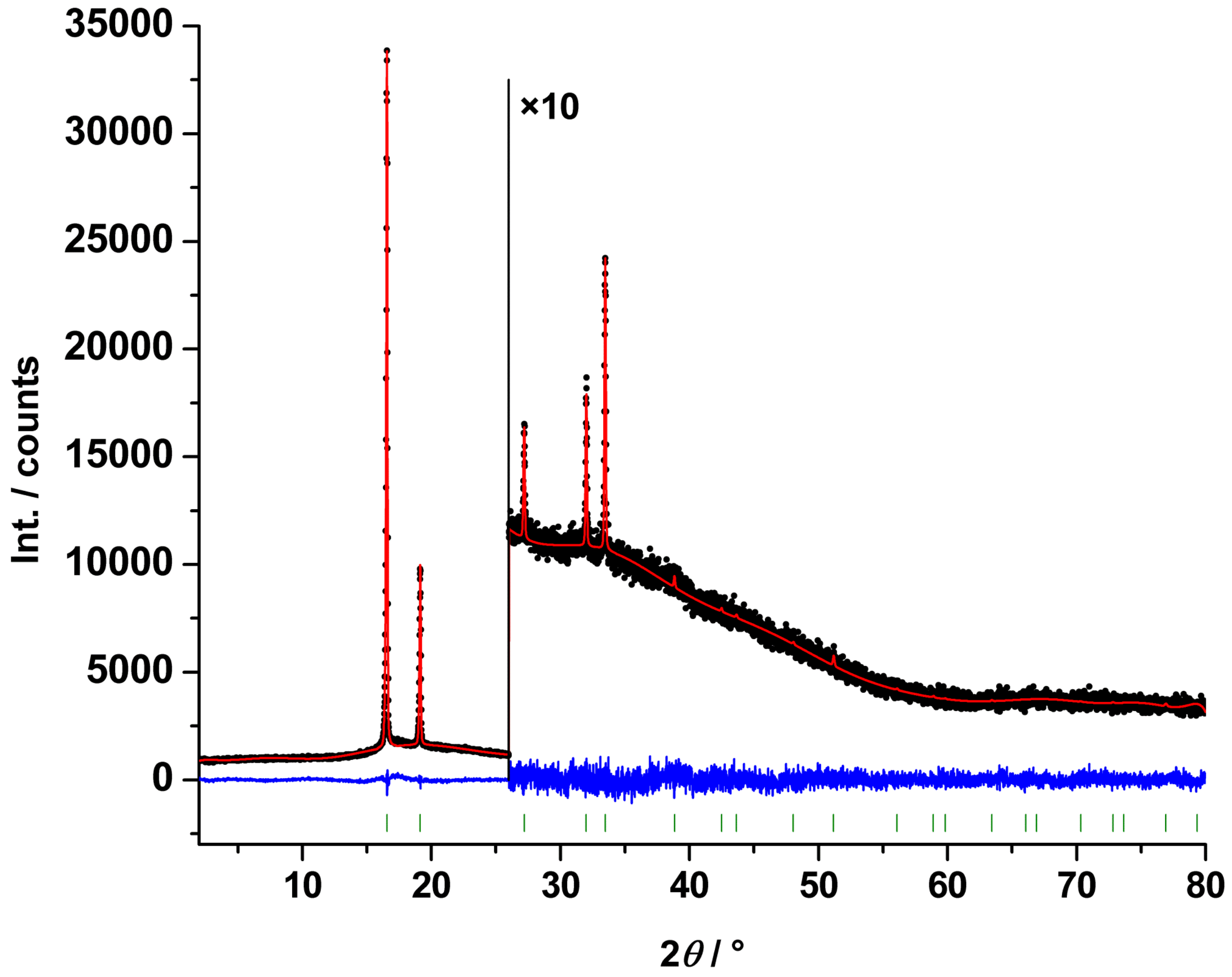

Figure S10 Pawley plot of **6**-B: observed (black), calculated (red) and difference (blue) profiles and tick marks (green).

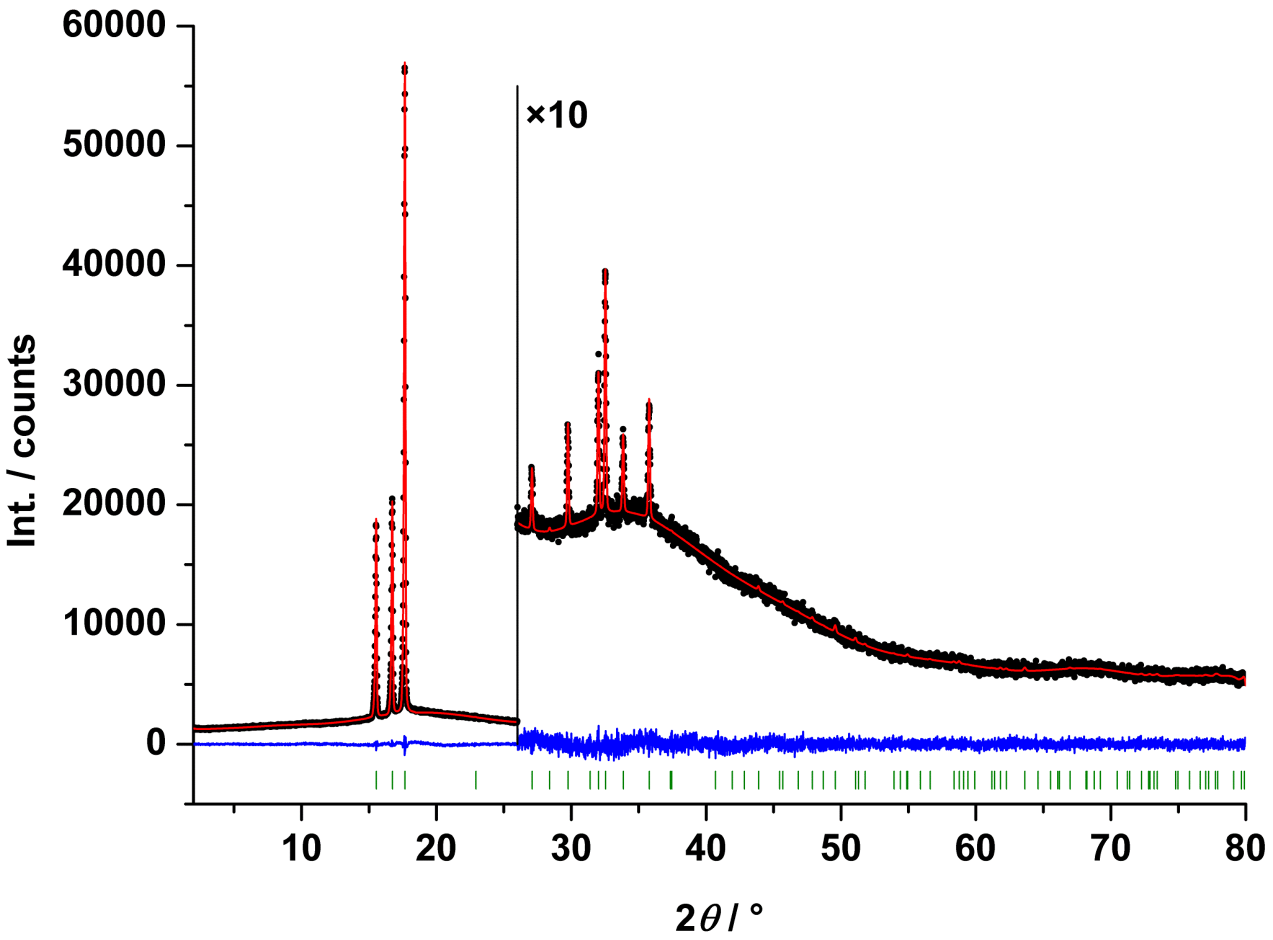

Figure S11 Pawley plot of **5**-B: observed (black), calculated (red) and difference (blue) profiles and tick marks (green).

S4. Stability of 7-C

XRPD measurements after one, two and three months revealed that **7**-C is not stable at room temperature and slowly transforms back to **7**-A (see Fig. S12). As shown in Fig. S12 there are four clearly free reflections (021 reflection at 15.4°, 111 at 17.4°, 102 at 18.7° and 112 at 20.1°) which indicate the increase of **7**-A in **7**-C over a period of three months. After one month a ratio of approximately 7:1 between **7**-C and **7**-A can be observed, after two months the ratio increases to 3:1 and after three months the ratio increases to 1:5. Therefore it can be assumed that after a longer period of time **7**-C converts back to **7**-A completely and that **7**-C is a meta-stable polymorph of **7**. Therefore no thermal effect could be observed during the cool-down process in the DSC (see Fig. 18 in the paper). Additional DSC and T-XRPD measurements on **7** up to 250 °C yielded only a partial conversion of **7**-A to **7**-C. Therefore the crystal structure of **7**-C was determined with a sample from DSC measurements heated up to 280 °C and cooled down to 20 °C.

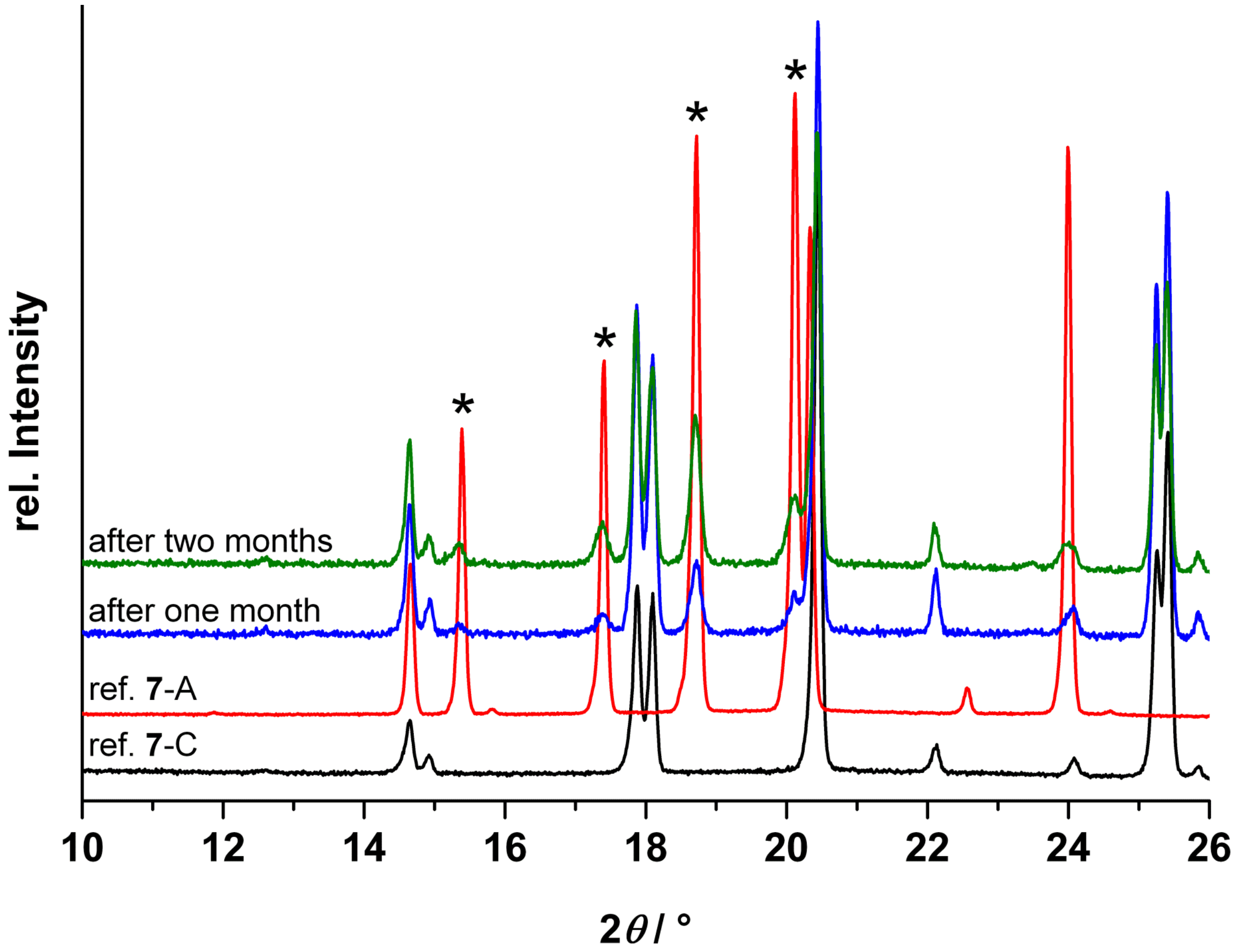

Figure S12 Overlay of the X-ray powder diffraction traces of myo-inositol, **7**, showing the reference trace of **7**-C (black) and **7**-A (red), **7**-C after one month (blue), two months (green) and three months (violet) stored at room temperature. Black asterisks indicate the reflections of interest.

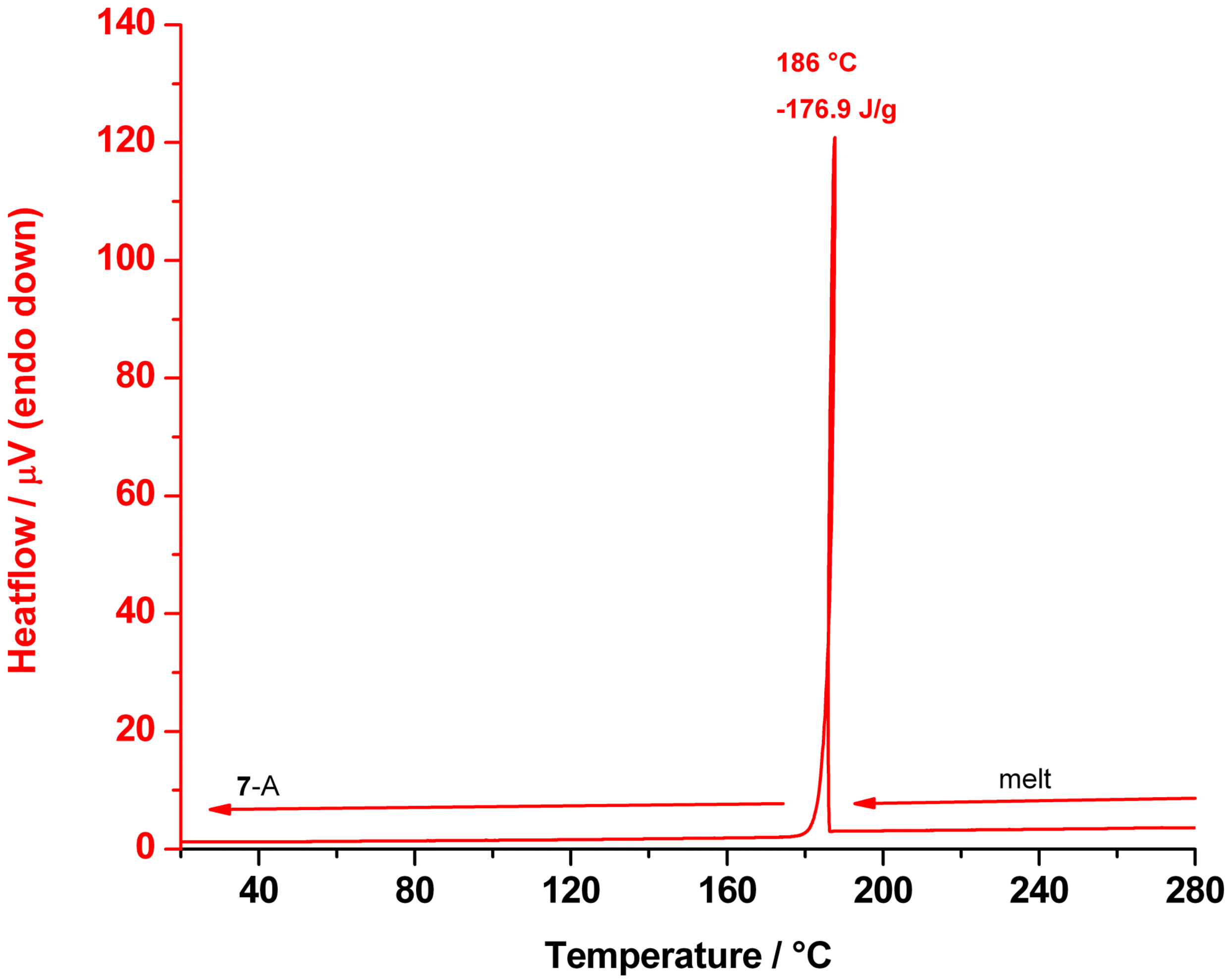

Figure S13 DSC trace of myo-inositol, **7**, measured from 280 down to 20 °C showing the recrystallisation of **7**-A from the melt at 186 °C.
